# Telomerase Is Required for Zebrafish Lifespan

**DOI:** 10.1371/journal.pgen.1003214

**Published:** 2013-01-17

**Authors:** Catarina M. Henriques, Madalena C. Carneiro, Inês M. Tenente, António Jacinto, Miguel Godinho Ferreira

**Affiliations:** 1 Instituto Gulbenkian de Ciência, Oeiras, Portugal; 2 Instituto de Medicina Molecular, Lisbon, Portugal; 3 CEDOC, Faculdade de Ciências Médicas, Lisbon, Portugal; Stanford University, United States of America

## Abstract

Telomerase activity is restricted in humans. Consequentially, telomeres shorten in most cells throughout our lives. Telomere dysfunction in vertebrates has been primarily studied in inbred mice strains with very long telomeres that fail to deplete telomeric repeats during their lifetime. It is, therefore, unclear how telomere shortening regulates tissue homeostasis in vertebrates with naturally short telomeres. Zebrafish have restricted telomerase expression and human-like telomere length. Here we show that first-generation *tert^−/−^* zebrafish die prematurely with shorter telomeres. *tert^−/−^* fish develop degenerative phenotypes, including premature infertility, gastrointestinal atrophy, and sarcopaenia. *tert^−/−^* mutants have impaired cell proliferation, accumulation of DNA damage markers, and a p53 response leading to early apoptosis, followed by accumulation of senescent cells. Apoptosis is primarily observed in the proliferative niche and germ cells. Cell proliferation, but not apoptosis, is rescued in *tp53^−/−^tert^−/−^* mutants, underscoring p53 as mediator of telomerase deficiency and consequent telomere instability. Thus, telomerase is limiting for zebrafish lifespan, enabling the study of telomere shortening in naturally ageing individuals.

## Author Summary

Telomerase mutations in humans give rise to premature ageing syndromes. In animals, the wealth of knowledge in telomere biology has been biased by the almost exclusive analysis of long-telomere mice. The role of telomere shortening requires investigation in organisms that, much like humans, have evolved telomere length as an internal cell division “timer.” We provide evidence for such a model. We show for the first time that telomerase is required during zebrafish lifespan. In contrast to mice, first-generation telomerase zebrafish mutants display degenerative phenotypes and die prematurely by one year of age. Furthermore, we show that most telomerase deficiency in this model leads to time- and tissue-specific apoptotic and senescence responses, highlighting different tissue thresholds to telomere dysfunction. Our results show that telomeres are maintained just above a critical threshold and that telomerase function is truly limiting for zebrafish lifespan and tissue homeostasis, closely mimicking the human scenario.

## Introduction

Telomeres constitute the ends of linear chromosomes, comprising DNA (TTAGGG)n repeats and its associated proteins, known as the shelterin complex [1]. Telomeres provide protection against erosion of chromosome-ends that occurs with each cell division as a result of the “end-replication problem” [2]. Additionally, they prevent the recognition of chromosome termini as deleterious DNA double strand breaks (DSBs). If this function fails, chromosome-ends induce DNA damage responses (DDRs) that comprise the activation of p53 [3]. Telomerase, a reverse transcriptase, counteracts chromosome-end depletion by elongating telomeres through the action of its catalytic unit (Tert) and RNA template (Terc) [4], [5]. Because telomerase expression is restricted in human somatic cells, telomeres shorten during our lifespan [6]. Human somatic cells lose around 100 base pairs of telomeres per population doubling [7], leading to a limit of about 50–80 cell divisions in culture, known as the Hayflick limit [8].

Impaired tissue homeostasis is at the core of several human diseases, including ageing-associated degeneration [9]. Premature ageing syndromes such as Werner, Hutchinson-Gilford and Dyskeratosis Congenita (DC) share the common trait of shorter telomeres, accelerated ageing and reduced lifespan [10]. DC, in particular, can be caused by mutations in the telomerase *tert* or *terc* genes, and there is a direct correlation between telomere length and disease severity [11].

Telomeres become dysfunctional due to critical shortening, oxidative damage or uncapping [12]. Dysfunctional telomeres induce DDRs characteristic of damage-induced DSBs [13]. Depending on the cell type, level of DNA damage and p53/p63/p73 status, dysfunctional telomeres initiate an apoptotic response or a G1 cell cycle arrest, leading to senescence [14]. While high levels of DNA damage are thought to trigger apoptosis via *puma* (p53 upregulated modulator of apoptosis) activation, low levels are most likely to cause cell cycle arrest via *p21* activation [14]. Telomere maintenance, therefore, dictates survival and replicative potential of cells, directing tissue homeostasis.

Most of our knowledge of vertebrate telomeres comes from inbred mice strains with long telomeres [15]. Several generations of intercrossing between telomerase deficient mice are needed before telomere shortening has a noticeable impact at the organism level [16]–[18]. Data from late generation telomerase knockout mice suggest that cell senescence [19] and/or apoptosis [20] play a critical role in the observed degenerative phenotypes. Either *puma* [21] or *p21* [19] deletion separately ameliorate degenerative phenotypes observed in late generation telomerase knock-out (KO) mice. Stem cell exhaustion via *puma*-mediated apoptosis is crucial in limiting the life span of late generation *terc* knockout mice [21]. Whether artificially shortening telomeres in the long telomere mouse strains or the use of other genetic backgrounds with shorter telomeres reproduces the way in which human tissues respond to telomere lifetime erosion remains an open question.

Recently, a wild-derived inbred mouse strain (Cast/EiJ) has been proposed as a better model for understanding telomere dysfunction in humans, given its shorter telomeres [22]. Telomerase deficiency in this strain gives rise to first generation defects similar to the ones observed in human DC syndromes [22]. Thus, telomere length may be limiting for Cast/EiJ longevity, making it a promising alternative to the current mouse models. However, molecular responses to dysfunctional telomeres in this model remain to be elucidated.

It is critical to investigate complementary vertebrate models to understand what is the most likely impact of telomere exhaustion in a biological system that, like humans, has evolved to have telomere length as an internal cell division “clock”. Zebrafish, a teleost fish that exhibits gradual senescence, is a promising vertebrate model for telomere biology. Contrary to the inbred laboratory mouse, zebrafish have heterogeneous telomeres of human-like length [23]. Despite detection of telomerase activity in various tissues, zebrafish telomeres shorten with age [24]. Like humans [6], telomerase expression in zebrafish somatic cells is not sufficient to prevent telomere shortening [24]. Telomere shortening was associated with impaired regenerative responses in the aged fish, denoting a role for telomere in homeostasis of adult tissues. Accordingly, a zebrafish mutant for the telomere repeat binding factor 2 (Terf2) accumulates senescence markers and this is accompanied by central nervous system necrosis and decreased survival [25].

Here we show that first generation telomerase deficient zebrafish have shorter telomeres than wild-type siblings and die prematurely. *tert^−/−^* fish are born and develop normally until adulthood, but progressively develop accelerated degenerative phenotypes characteristic of disrupted tissue homeostasis. These include premature infertility, gastrointestinal atrophy, loss of body mass, increased inflammation and sarcopaenia at terminal stages. Underlying these phenomena is a sustained decrease in cell proliferation, an acute apoptotic response and accumulation of DDR foci. Removal of p53 function rescues cell proliferation, but not apoptosis, in high turnover tissues, such as testes and gut. This implicates p53 as a critical mediator of telomerase-dependent proliferative defects observed in *tert^−/−^*. Thus telomerase and consequent telomere shortening play a key and limiting role in tissue maintenance during a zebrafish lifespan.

## Results

### Telomerase mutant zebrafish have shorter telomeres

In order to examine the consequences of telomerase depletion in zebrafish, we used the currently available but yet uncharacterized, *tert* ^hu3430^ line produced by ENU-*tilling* screen at Utrecht University, Netherlands [26]. This telomerase mutant line carries a T→A transition in the second exon of the *tert* gene giving rise to an early stop codon. For simplicity, we will refer to the *tert* ^hu3430^ homozygous mutant strain as *tert^−/−^*.

**Figure 1:**
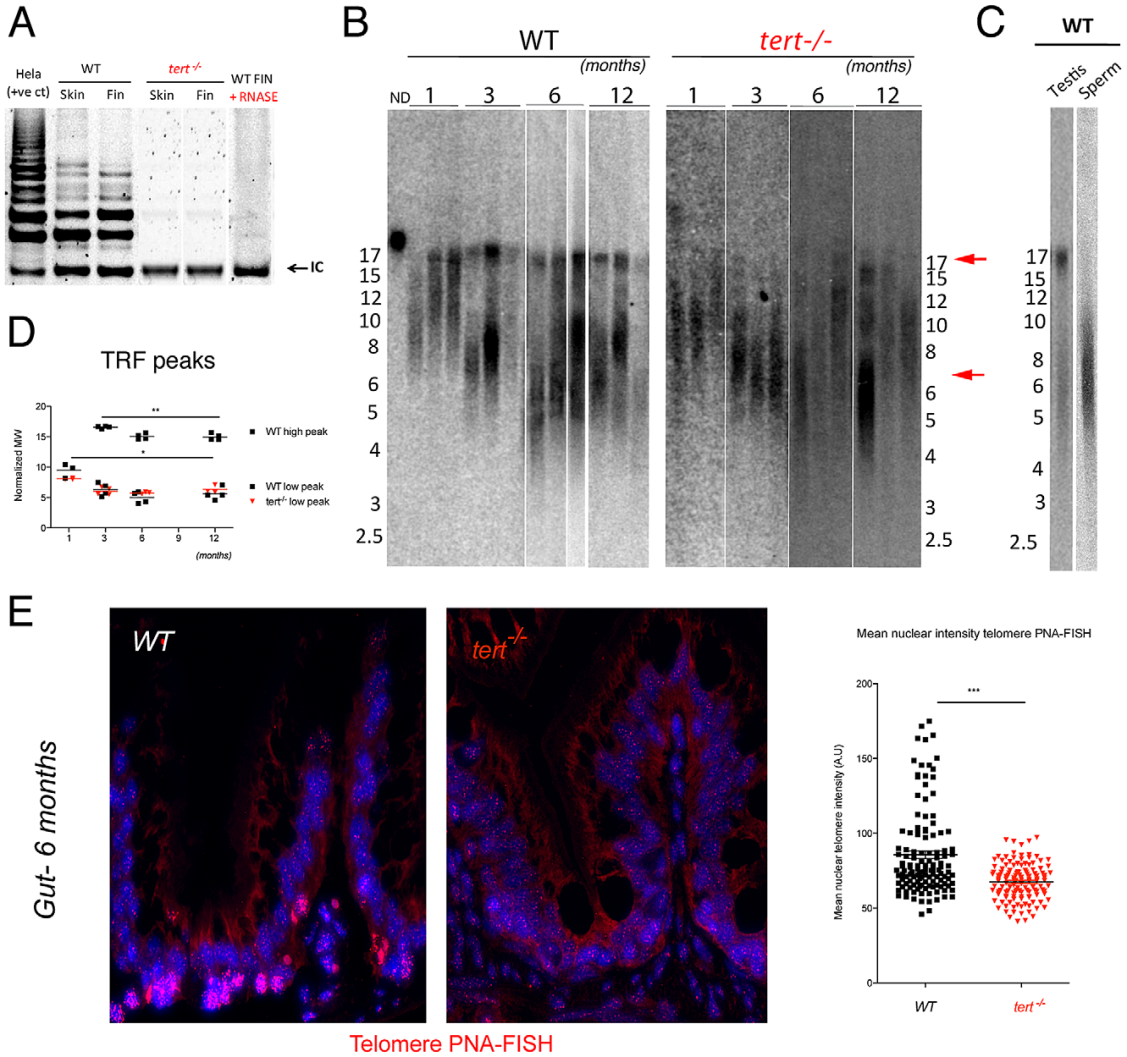
Telomerase mutant zebrafish have shorter telomeres than WT siblings. A) Representative image of TRAP assay showing that telomerase is not active in the *tert^−/−^* zebrafish, as compared to *tert^+/+^* siblings. Here shown are caudal fin and skin protein extracts. Hela cell extract is shown as positive control. N = 4. B) Representative image of restriction fragment analysis of caudal fin genomic DNA of 3 different individuals at different ages, by southern blot (random primer-labelled telomeric probe (CCCTAA)_12_^32^P-dCTP). *tert^+/+^* Zebrafish have heterogeneous telomeres, with two distinct peaks of different lengths. In *tert^+/+^* the highest peak (∼16 Kb, top red arrow) becomes more distinct after 1 months of age and decreases in length over-time (B and D). The lowest peak of telomere intensity also decreases in length (bottom red arrow, B and D). *tert^−/−^* zebrafish have shorter telomeres than *tert^+/+^* siblings in different tissues (see also Figure S1A and S1B), observed by the decrease in length of the higher TRF peak. The shortest TRF peaks accompany those of *tert^+/+^* siblings, and decrease over-time at similar rates. C) Testes fractionation in *tert^+/+^* reveals the two-telomere length populations in whole testes, whereas mature sperm only shows the shorter TRF smear of about 6 Kb, suggesting different telomere lengths in different cells within a tissue. D) TRF mean sizes were calculated as described in [50]. E) Telomere PNA-FISH in 6-month-old gut tissue shows cells with different telomere intensities in the wild type, mainly localizing to the proliferative niche. In contrast *tert^−/−^* mutants display cells with less bright and more homogeneous telomere intensity.

To test whether *tert* ^−/−^ mutants had a functional telomerase, we performed the commonly used Telomere Repeat Amplification (TRAP) assay [27]. We observed no amplification bands corresponding to telomere elongation in the TRAP assay as compared to *tert^+/+^* controls (Figure 1A), indicating that *tert^−/−^* mutants lack active telomerase. The consequence of absence of telomerase is continuous telomere shortening. Accordingly, Telomere Restriction Fragment (TRF) analysis by Southern blot revealed a significant reduction in average telomere size (Figure 1B and 1D). This attrition was highlighted by the significant reduction in intensity of the higher molecular weight TRFs (∼16 Kbp), as compared to *tert^+/+^* siblings (Figure 1B), in all tissues tested (Figure S1). Additionally, a lower molecular weight TRF population of approximately 6 Kbp is present in both *tert^+/+^* and *tert^−/−^* (arrows in Figure 1B and 1D and Figure S1). Zebrafish telomere sequences are exclusively terminal, as all TRF signal disappears after BAL-31 (5′- and 3′-exonuclease) digestion (Figure S1C).

The presence of two telomere populations in *tert^+/+^* zebrafish is suggestive of restricted telomerase activity. Accordingly, the lower population of TRFs shortens over-time both in *tert^+/+^* and *tert^−/−^* (Figure 1B, 1D, 1E). We also observe discrete but significant shortening of the higher TRF population in *tert^+/+^*, suggesting that telomerase activity is not sufficient to prevent telomere loss. This bimodal TRF pattern was observed in all *tert^+/+^* tissues except for blood, where we detect a single TRF of higher molecular weight (∼16 kbp; Figure S1A, S1B) and in sperm, where we observed a single TRF signal of approximately 6 Kbp (Figure 1C).

Consistent with our TRF analysis, we observe the presence of cell populations with different telomere intensities by telomere-PNA FISH (Figure 1E). This technique clearly shows that *tert^+/+^* tissues, such as the gut, are composed of cells with different telomere intensities. Cells with high intensity in telomere signal localize primarily to the base of the villus, suggestive of being early precursor cells. In contrast, *tert^−/−^* tissue shows more homogeneous, low intensity, telomere-PNA FISH. Such pattern mirrors our TRF results of high and low telomere lengths and suggests that telomerase expression is restricted to certain cells types as observed in humans [6].

### Telomerase zebrafish mutants die prematurely of body wasting

**Figure 2:**
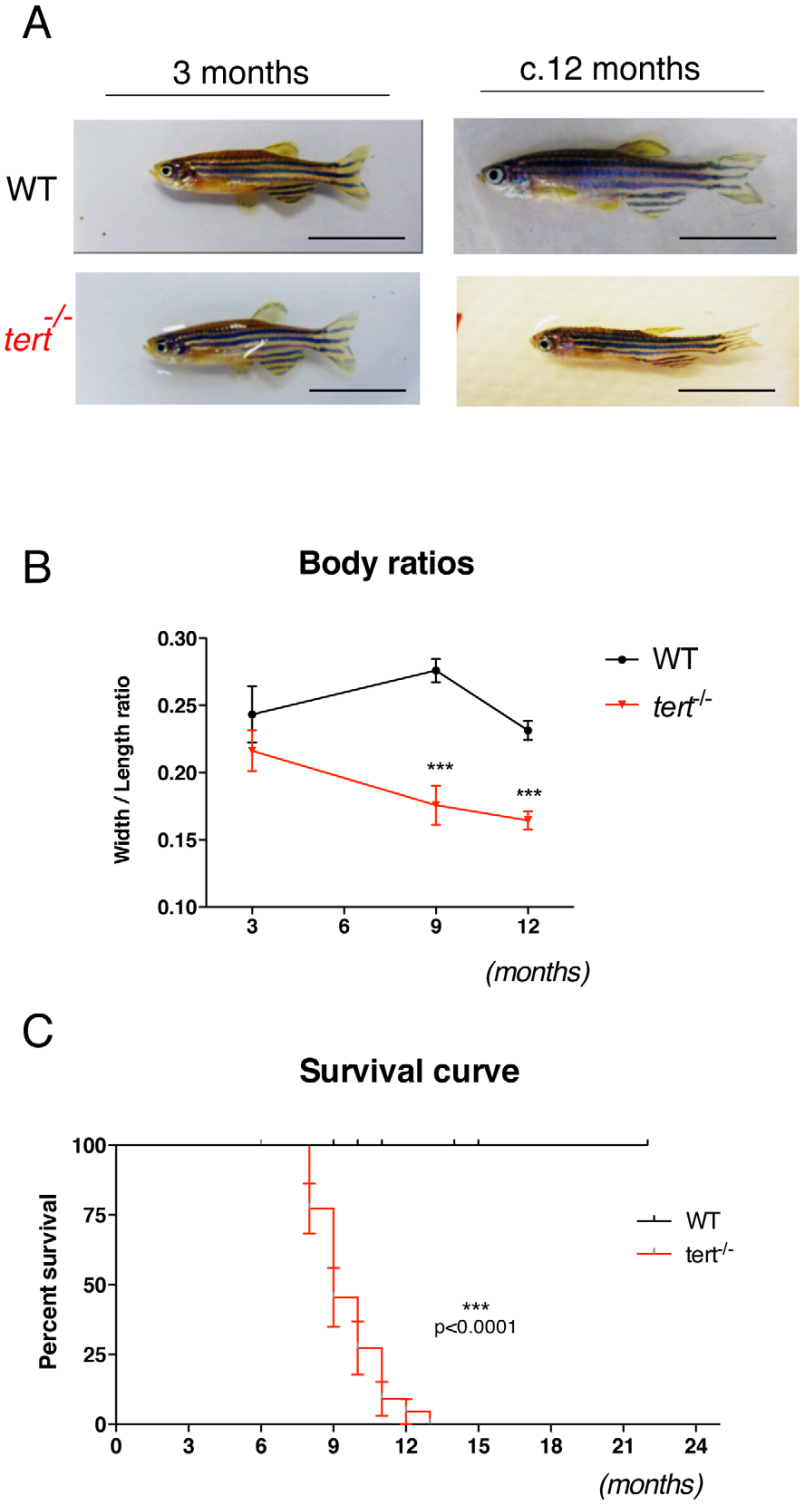
First-generation telomerase mutant zebrafish show progressive body wasting and die prematurely. A) Representative images of *tert^+/+^* and *tert^−/−^* zebrafish show that *tert^−/−^* fish are born and develop normally until reproductive maturity at ∼3 months of age, but progressively lose body mass since then, B) represented as an overall reduction in width/length ratios as compared to wild-type siblings N≥6 p<0.001. This progressive wasting phenotype is accompanied by increase in mortality. C) Kaplan-Meier curve showing that *tert^−/−^* zebrafish have significantly reduced survival when compared to *tert^+/+^* siblings (AVG lifespan 9 *versus* >22 months (p<0.005)). N = 24 *tert^−/−^*; N = 45 *tert^+/+^*. Data are represented as mean +/− SEM. Scale bar = 1 cm.

First generation *tert^−/−^* mutants, resulting from a heterozygous incross, are born healthy and develop past sexual maturity without any obvious defects (Figure 2A). From 4–6 months onwards, we observed a consistent gradual decrease in body mass reflected in declining width/length body ratios (Figure 2B). This wasting phenotype was observed in all *tert^−/−^* individuals at their time of death (n = 24). In contrast, we were unable to detect wasting in any of the *tert* ^+/+^ siblings (n = 45) until the end of the experiment (22 months). Wasting (also known as cachexia) is a common phenotypic alteration in aged organisms, including humans, and is usually associated with muscle sarcopaenia and frailty syndromes [28].

Progressive wasting in *tert^−/−^* fish was accompanied by an increase in mortality (Figure 2C). *tert^−/−^* mutants die significantly earlier than their *tert* ^+/+^ siblings (average lifespan of 9 *versus* >22 months, p<0.005). Due to a male sex bias in our crosses that affected all *tert^+/+^*, *tert^+/−^* and *tert^−/−^* progeny, we were unable to obtain significant numbers for female analysis and so the data hereafter presented reflects the study of males alone. Sex determination in zebrafish is still largely unknown, but it is thought to be highly influenced by environmental factors [29].

### Telomerase depletion produces a time- and tissue-specific degeneration

**Figure 3:**
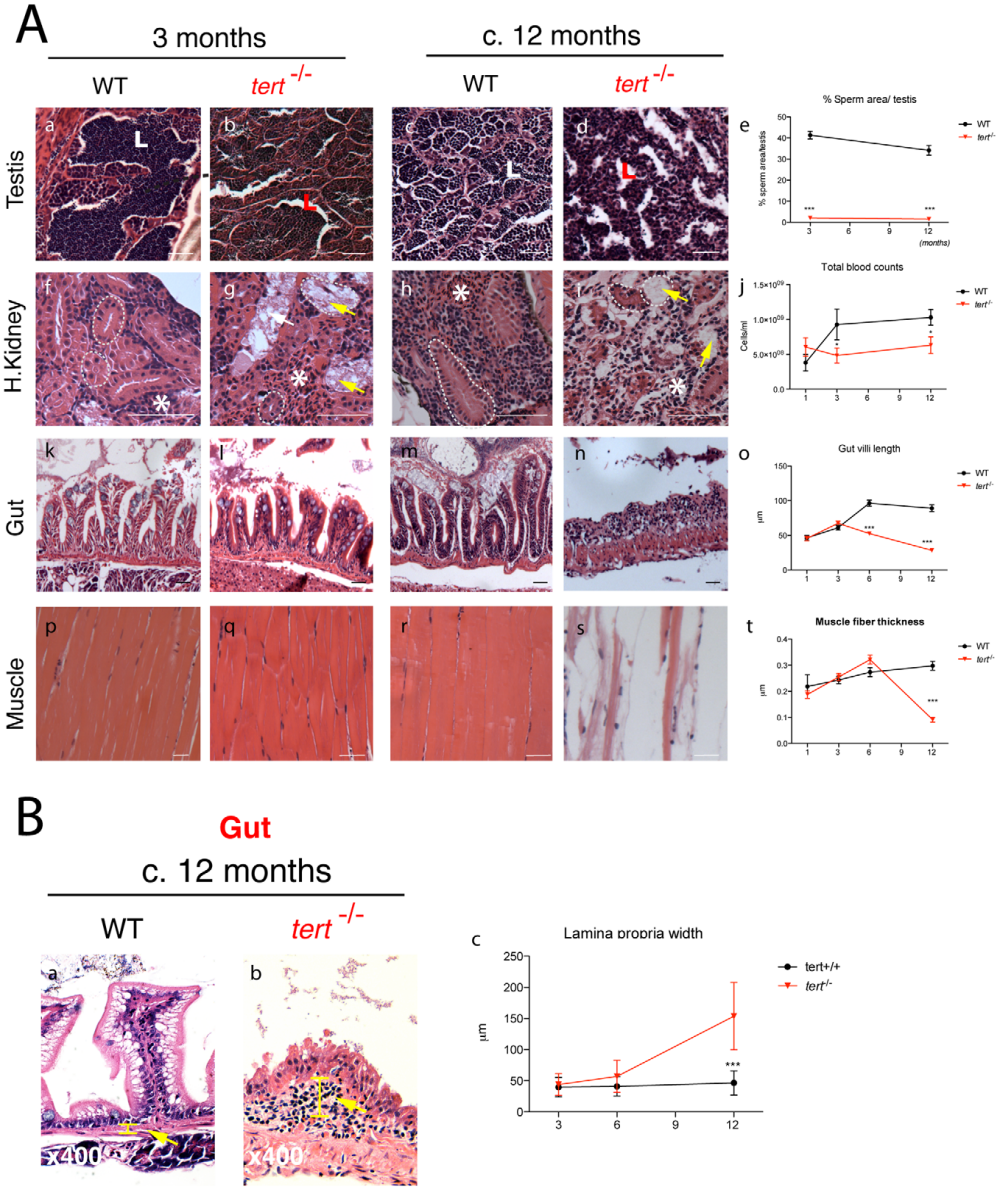
Telomerase depletion leads to a time- and tissue-dependent degeneration. A) Representative images of tissue sections of *tert^−/−^* Zebrafish and *tert^+/+^* siblings, stained with hematoxilin-eosin. *tert^−/−^* zebrafish show progressive tissue deterioration. Severe histological abnormalities are first evident in proliferative tissues (testes, gut and head kidney marrow) and later in non-proliferative (muscle). *tert^−/−^* Zebrafish show reduced sperm in testes lumen (L) (Ab and d; p<0.001). The head kidney shows progressive defects in the marrow area (white asterisks) (Ag and i, which correlates with a decrease in total blood (p = 0.0228) cells when compared to *tert^+/+^* siblings from an early age (3 months, N≥5) (Aj). Mesonephric tubules in the head kidney also degenerate in *tert^−/−^* (Ag and i dashed outlines). Gut atrophy in *tert^−/−^*, reflected as decreased villi length (Al, n, o), becomes significant from the age of 6 months (p<0.001). Muscle fibres are significantly thinner (p<0.001) at terminal time-points (c.12 months) (Aq, s, t, dashed outline); N≥5. B) *tert^−/−^* display progressive thickening of gut lamina propria, indicative of inflammation (Bb, c, yellow bar and arrow, N≥4). Data are represented as mean +/− SEM. Scale bar = 50 µm.

**Figure 4:**
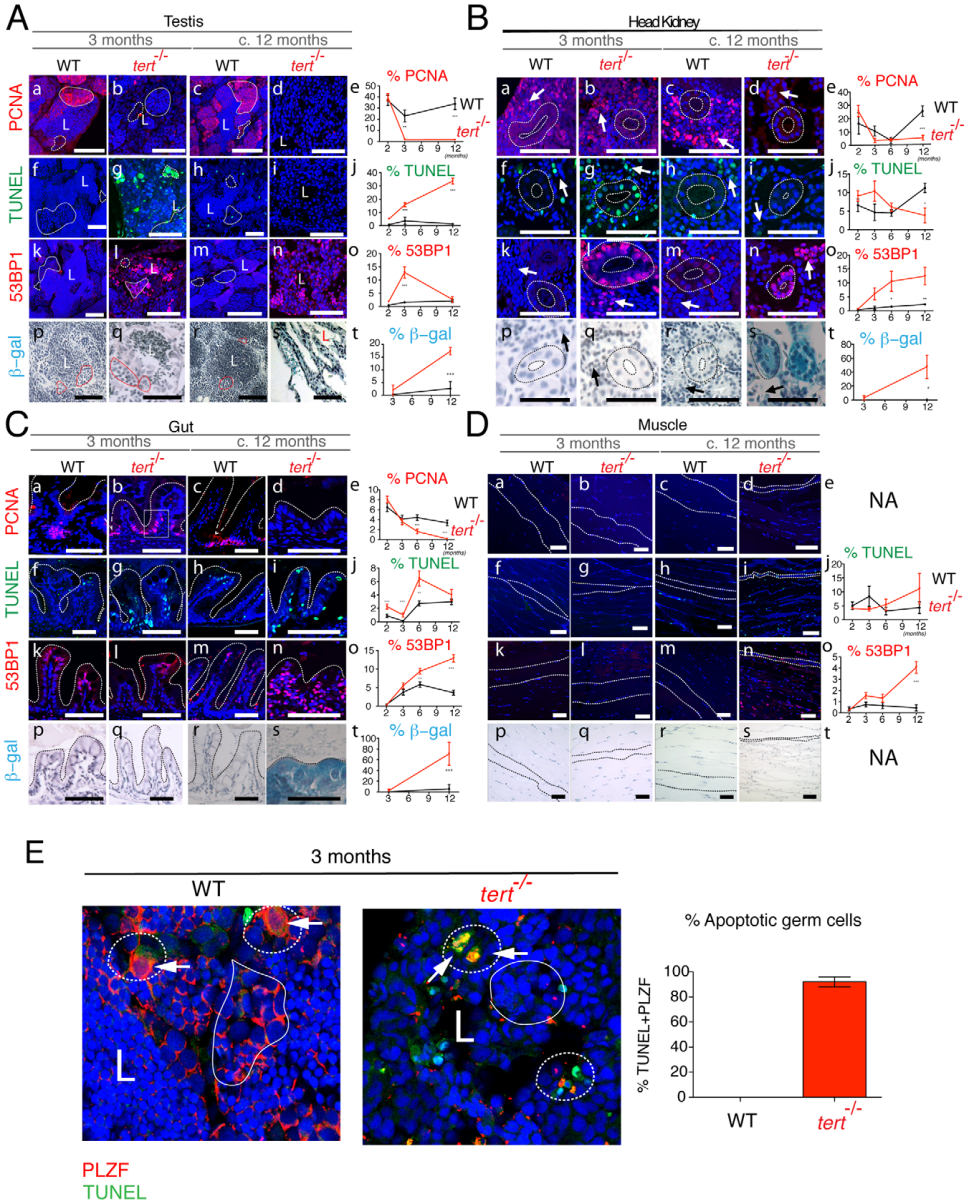
Proliferative tissue degeneration is accompanied by a sustained decrease in proliferation, acute apoptotic responses, and progressive accumulation of DDR foci. Representative immunofluorescence images of tissue sections in F1 *tert^−/−^* and *tert^+/+^* zebrafish show levels of proliferation (PCNA), apoptosis (TUNEL), DNA damage (53BP1) and senescence-associated β–galactosidase at the ages of 3 to c.12 months. Proliferative tissues such as A) testes, B) head kidney and C) gut sections show sustained significant decrease in proliferation in *tert^−/−^* as compared to *tert^+/+^* siblings (panels b, d and e) (p<0.001) and an acute apoptotic response at 3 months of age (p<0.001), which clears by c. 12 months (panels g, i and j). This is accompanied by a progressive increase in 53BP1 foci, reaching maximum significance at c. 12 months (panels l, n and o; p<0.001). This coincides with the presence of senescence-associated β –galactosidase at c.12 months (panels s and t). Note in the testes that most of apoptosis (TUNEL) seems to localize to the spermatogenic zone (Ag, dashed outline) and panel E, where we see an increase in TUNEL-labelled germ cells, labelled with the specific marker PLZF. Most of DNA damage (53BP1) locates to the proliferative zone of maturing spermatocytes (Al, uniform outline). Note in the head kidney B), both the proliferative haematopoietic tissue (Ba–d, arrows) and the non-proliferative mesonephric tubule epithelium (Ba–d, dashed outline) are affected by increased apoptosis (Bg, I, j), DNA damage (Bl, n and o) and senescence (Bs and t) in the *tert^−/−^*. D) Muscle, a largely non-proliferative tissue (Da–d) shows significant accumulation of DNA damage foci at in *tert^−/−^* by the age of c.12 months (Dn and o; p<0.001), when the muscle fibres are already atrophic (Dn, dashed outline). Quantifications were performed in at least 3 different fields of view of at least 3 different individuals of each genotype at the different time-points indicated in the graphs. Gut IF quantifications were calculated as number of positive cells per “crypt” zone (C) uniform square outline exemplified). Other tissues' IF was quantified as overall % positive cells. β-galactosidase was quantified as % area stained blue, per field of view. Data are represented as mean +/− SEM. Scale bar = 50 µm.

Histopathological analysis of different tissues revealed important phenotypic alterations in *tert* ^−/−^ mutants. Telomere shortening has been shown to affect primarily high turnover tissues in both humans [10] and late generation *terc* KO mice [30]. Accordingly, we noticed an order of events during tissue atrophy of *tert^−/−^* zebrafish. *tert^−/−^* zebrafish testes were the first to depict histopathological abnormalities; second were the liver, intestine, gills and pancreas; the third to be affected was the kidney and remaining organs, including the muscle (Figure 3, Figure 4 and summarized in Table S2).

Testes of *tert^−/−^* zebrafish show a severe imbalance, as early as 3 months of age, in size and ratio of the main spermatogenic classes: spermatogonia, spermatocytes and spermatids [31]. There was an atrophy of the differentiating and maturing spermatogenic stages (Figure 3Ab and 3Ad). This atrophy is consistent with what was observed in spermatogonia in the late generation *terc* KO mice [30] and *tert* KO mice [18], [32]. A consequence of these alterations is the significant decrease in mature sperm volume observed in the lumen of seminiferous tubules of *tert^−/−^* male fish (L in Figure 3Ab and 3Ad). Consistently, we observed that *tert^−/−^* zebrafish males are prematurely infertile (Figure S2). The scarce progeny originating from a *tert^−/−^* incross was not viable and displayed embryonic deficiencies consistent with lack of cell proliferation, such as failure to close the neural tube and body truncations (Figure S2). Female *tert^−/−^* mutants were initially fertile (Figure S2) but became infertile later in life, when body wasting became apparent (data not shown).

Similar to telomerase deficiencies in humans and mice with critically short telomeres, *tert^−/−^* zebrafish display blood defects, translated into a mild but significant decrease in total blood cells (Figure 3Aj). Accordingly, histopathological analysis of the head kidney marrow (the major hematopoietic organ in fish) revealed a trend towards depletion of the hematopoietic compartment, particularly at late time points (asterisks in Figure 3Ai). Other proliferative tissues, such as the gut, show a progressive decrease in microvilli length (Figure 3Al,n,o) and increased inflammation of the *lamina propria*, significant after the age of 6 months (Figure 3Bc). These changes progress into severe gut degeneration (necrotizing enteritis), most visible at terminal stages (Figure 3An). During this period, we observed a pervasive mucosal thickening (sloughing; bar in Figure 3Bb), denuded villous tips, and inflammatory cell infiltration (arrows in Figure 3Bb). Atypical intestinal epithelium compatible with severe dysplasia was also observed (data not shown).

Low-proliferative tissues such as the muscle (Figure 3Ap–t and Figure 4Dp–t) and liver (data not shown) only exhibit obvious degeneration at the latest time points of the *tert^−/−^* zebrafish lifespan. We observed acute and significant muscle degeneration (sarcopaenia) at terminal stages (Figure 3As) consistent with the wasting phenotype (Figure 2A and 2B). These observations suggest that low-proliferative tissues are also targets of telomere dysfunction, as has been suggested before in mice models [33]–[35]. Whether this happens in a cell autonomous or non-autonomous manner remains to be clarified.

Severe gut degeneration (necrotizing enteritis) is a prime candidate for cause of death of *tert^−/−^* zebrafish. This could account for mal-nutrition, consequent loss of muscle and wasting. Consistently, exocrine pancreas of 12 month-old *tert^−/−^* zebrafish lacked large numbers of bright secretory granules, characteristic of actively feeding fish (Table S2). Lastly, we did not observe neoplastic changes in any of the tissues studied.

### Tissue atrophy is preceded by lack of cell proliferation, apoptosis, and senescence

Telomere depletion was shown to affect primarily proliferative tissues with a high cell turnover. Quantification of cell division in *tert^−/−^* zebrafish using the S-phase marker PCNA revealed an overall decrease in cell division in proliferative tissues (Figure 4A–4C, panels a–e). This was particularly clear in the spermatogenic zone of the testes (filled-line outline in Figure 4Ab,d). Spermatogenesis is initiated by the proliferation of stem cells (Spermatogonia A and progenitor stem cells - Spermatogonia B). These are responsible for the continuous renewing of this highly proliferative tissue. Decrease in cell proliferation in the testis was accompanied by an initial burst in apoptosis, as detected by TdT-mediated dUTP nick end labelling (TUNEL; dashed line outline in Figure 4Ag). This burst of apoptosis in *tert^−/−^* is restricted to the germinal centres, as shown by co-localization with the specific germ cell marker PLZF [36] (Figure 4E), explaining why tissue homeostasis is compromised in these animals, as shown in mice [37]. Initial increase of apoptosis was followed by a progressive decline, losing statistical significance at later time points (Figure 4Ag,i,j).

A second player in disrupting tissue integrity is the accumulation of senescent cells [9], [20], [38]. We observed a progressive increase in cells presenting strong 53BP1 *foci* in *tert^−/−^* testes, indicative of persistent or irreparable DNA damage (Figure 4Al,n,o and Figure S3d). Persistent 53BP1 has been described as a hallmark of cell senescence, accumulating preferentially at telomeres [39], [40]. Consistently, we observed an increase in senescence-associated β-galactosidase (SA-β-gal) staining at later time points (Figure 4As,t).

Similar to testes, both kidney marrow and gut show an initial up-regulation of apoptosis, statistically significant at 3 months old (Figure 4Bg,j and 4Cg,j). The head kidney in zebrafish has a dual function of excretion and haematopoiesis [41]. Apoptosis is up regulated both in the kidney mesonephric tubules and haematopoietic tissue (dashed outline and arrow, respectively, in Figure 4Bg), consistent with the decreased blood cell levels observed (Figure 3Aj). In the gut, this increase in apoptosis is accompanied by decreased proliferation at the base of the villi, statistically significant from 6 months onwards (Figure 4Ca–e). Decreased cell proliferation in the gut is accompanied by progressive increase in DNA damage *foci*, as detected by 53BP1 staining (Figure 4Ck–o and Figure S3b). In all proliferative tissues, 53BP1 staining reached its peak at terminal stages, where no apoptosis is detected (Figure 4A–4C, panels n,o). Senescent cells have been described to be resistant to apoptosis [42]. Consistently, in proliferative tissues, we noticed that 53BP1 foci containing cells corresponded to regions where SA-β-gal staining was evident (panels n and s in Figure 4A–4C).

Low-proliferative tissues, such as the muscle, only show significant defects at later time points. Muscle sarcopaenia is accompanied by an acute increase in cells stained with 53BP1 (Figure 4Dn,o). Contrary to proliferative tissues, 53BP1 staining in the muscle (Figure 4Dn) did not correlate with an increase in cell senescence, as we were unable to detect significant SA-β-gal staining (Figure 4Ds,t).

### p53 expression leads to *puma*-apoptosis and *ccng1*-senescence

**Figure 5:**
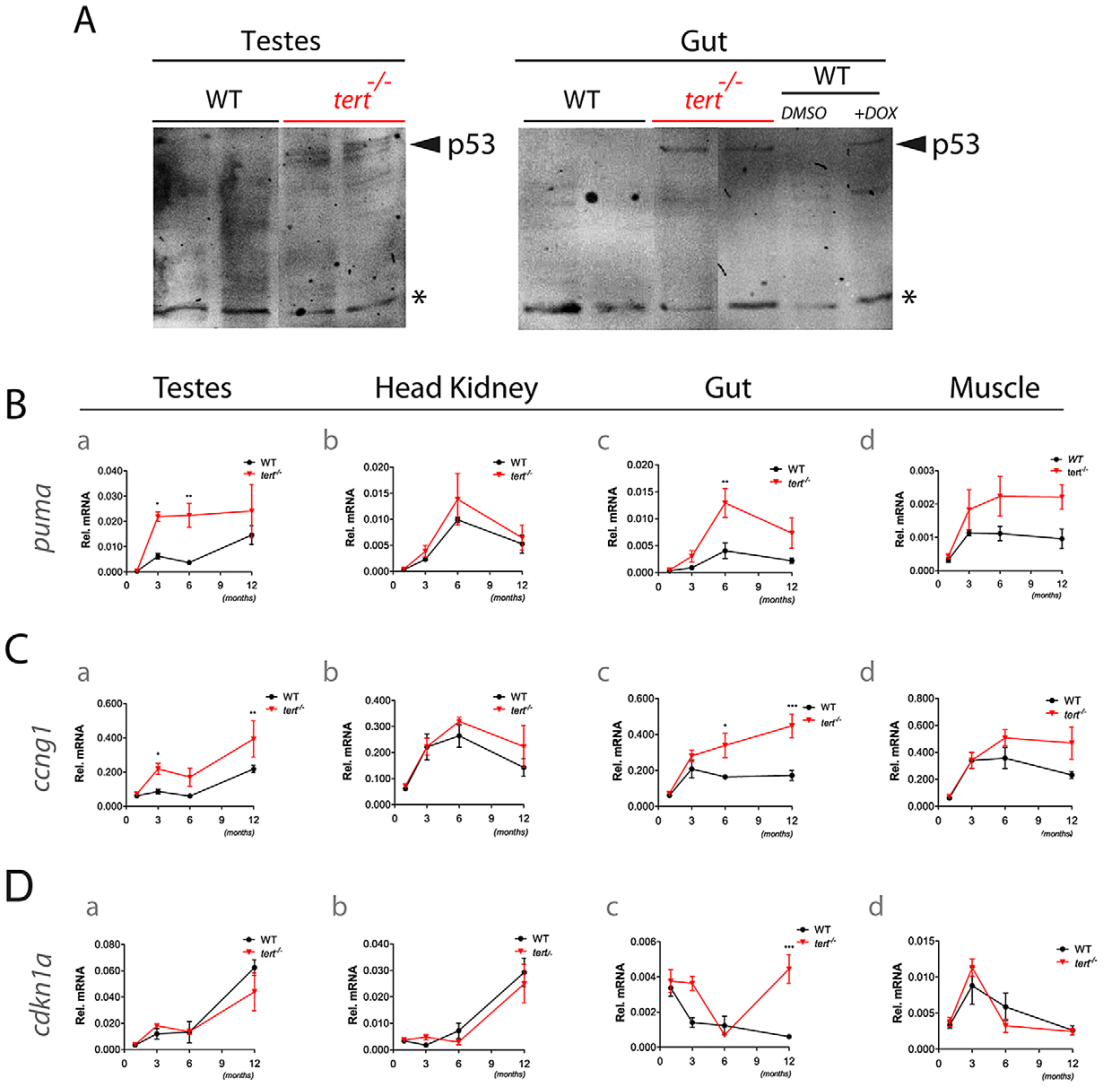
Tissue degeneration is accompanied by p53 induction with a *puma* acute response and sustained increase of *cyclin G1* and *cdkn1a* expression. A) Immunoblot analysis of p53 in 10-month old WT and *tert^−/−^* testes and gut lysates. 6 month-old WT zebrafish were injected with the DNA damaging agent doxorubicin to serve as positive control for p53 activation. Asterisk depicts a non-specific cross-reactive band that serves as loading control. RT-qPCR analysis showing expression of B) pro-apoptotic (*puma*) and cell cycle arrest targets (C) *cyclin G1* and D) *cdkn1a*) in testes, head kidney, gut and muscle of 1, 3, 6 and c.12 months old WT and *tert^−/−^* zebrafish (N = 3 to 8 fish per genotype). Data are represented as mean +/− SEM. *Rel. mRNA* refers to relative mRNA levels of each gene normalized to *beta-actin*.

In late generation telomerase KO mice, telomere shortening activates a p53 dependent DDR that culminates in apoptosis and cell-cycle arrest [21]. Similarly, we observed a p53 response in proliferative tissues of *tert^−/−^* zebrafish, such as the testes and gut (Figure 5A). This response is accompanied by an acute up-regulation of *puma* (Figure 5B a, c). Consistent with our apoptosis data, *puma* expression is reduced at later stages (c.12 months of age; Figure 5B a, c). In the gut, *puma* expression is replaced by sustained up-regulation of the cell cycle arrest targets *cdkn1a and cyclin G1* (Figure 5Cc and 5Dc). Recently, *cyclin G1* has been depicted as a p53-dependent target in zebrafish [43]. Consistently, up-regulation of cell cycle inhibitors through p53 activation led to G1 arrest and cell senescence. This has been described as recurrent phenomena in telomerase knockout murine tissues with dysfunctional telomeres [19]. Finally, we do not detect a significant p53 response in either the head kidney (an heterogeneous tissue), or in the muscle, a low-proliferative tissue.

### Elimination of *tp53* function partially rescues *tert^−/−^* phenotypes

**Figure 6:**
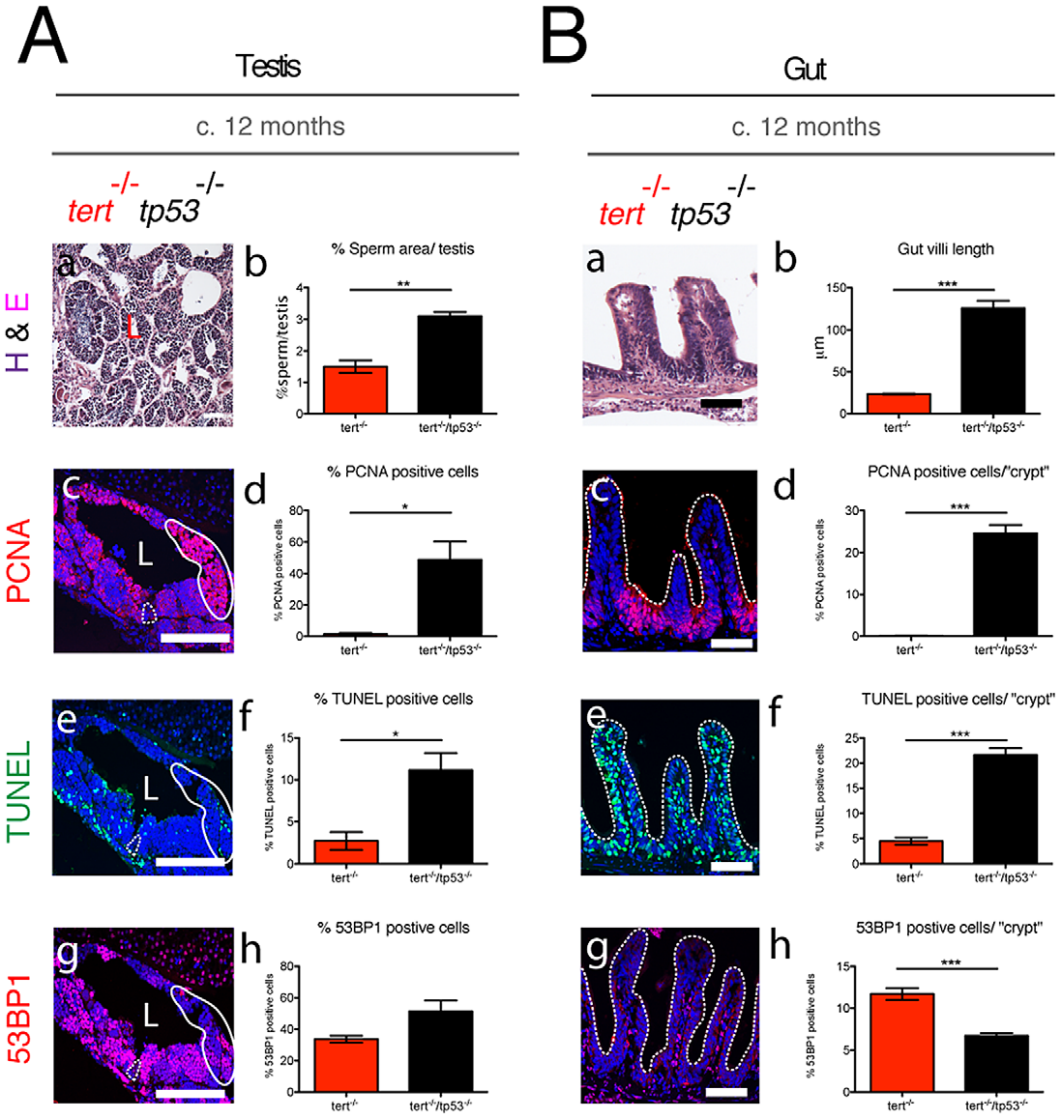
Elimination of *tp53* function partially rescues *tert^−/−^* degeneration in proliferative tissues. *tert^−/−^tp53^−/−^* show increased proliferation and apoptosis in both testes (Ac, d and e, f) and gut (Bc, d and e, f), as compared to *tert^−/−^* alone. In the testis, dashed outline represents spermatogenic zone, uniform outline proliferative zone of maturing spermatocytes and L the lumen where mature sperm is located. Elimination of *tp53* function partially rescues mature sperm numbers (Aa, b) but completely rescues gut villi length (Ba, b). DNA damage as assessed by 53BP1 is maintained in *tert^−/−^tp53^−/−^* testes (Ag and h) and decreased in the gut (Bg and h), as compared to *tert^−/−^*. N≥3. Data are represented as mean +/− SEM. Scale bar = 50 µm.

Our data show that telomerase deficiency in zebrafish gives rise to a p53 response, which ultimately culminates in cell cycle arrest (Figure 5), as observed in mice models [19]. Accordingly, *tp53* deletion in mice was shown to ameliorate late generation *tert* KO phenotypes such as infertility [44]. To test whether p53 could be mediating the phenotypes observed in the *tert^−/−^* zebrafish, we crossed *tert^+/−^* with *tp53^−/−^* mutants (*tp53 ^zdf1/zdf1^* [45]) to produce double mutant *tert^−/−^tp53^−/−^* zebrafish. Mortality was significantly rescued, since at c.12 months, approximately 87% of *tert^−/−^ tp53^−/−^* (N =  31) were alive, compared to 0% of *tert^−/−^* (N = 24). Immunofluorescence analysis in *tert^−/−^ tp53^−/−^* proliferative tissues, revealed a dramatic rescue of cell proliferation in tissues that were most affected in *tert^−/−^* (testes and gut at c.12 months; Figure 6Ac, d and 6Bc, d).

This rescue in cell proliferation, however, was not sufficient to prevent testis atrophy and decreased number of mature sperm in *tert^−/−^ tp53^−/−^* (Figure 6Aa,b). In contrast, rescue of the proliferative capacity in the gut of the double mutant clearly suppressed *tert^−/−^* gut villi length defects (Figure 6Ba,b). Despite an increased cell proliferation, this partial phenotypic rescue was accompanied by a significant increase in TUNEL-labelled cells in the *tert^−/−^ tp53^−/−^* testis (Figure 6Ae, f) and gut (Figure 6Be, f) and maintenance or slight decrease in 53BP1 staining (Figure 6Ag, h and 6Bg, h). Thus, similar to the mouse model [44], p53 appears to mediate *tert^−/−^* phenotypes, as *tp53* function depletion partially rescues or delays proliferative tissue degeneration observed in *tert^−/−^* zebrafish.

## Discussion

Most of our knowledge on how vertebrates respond to short telomeres derives from laboratory mice, which are particularly different from humans not only in what respects telomere length, but also in cell immortalization and entry into senescence [46]. Zebrafish has recently emerged as an attractive complementary vertebrate model for studying telomere biology. Zebrafish possess short telomeres that decline with age and correlate with impaired tissue repair [24]. These observations motivated us to investigate the impact of telomere shortening in a biological system that, like humans, may have evolved to use telomere length as an internal cell division “clock”.

In our current work, we show that zebrafish telomerase mutants have premature degenerative phenotypes and decreased lifespan in the F1 generation. This contrasts with results obtained from telomerase KO mice, where degenerative phenotypes are only observed upon several generations of null crosses [16]–[18], [32]. The phenotypes we observe correlate well with the accumulation of short telomeres in *tert* ^−/−^ zebrafish, along with the disappearance of long telomeres. The two telomere populations may reflect different phenomena: 1) Presence of undigested genomic DNA; 2) Distinct populations of sub-telomeric sequences in the same cells or 3) Telomeres of different lengths present in distinct cells. We excluded the first two possibilities. Regarding the first point, undigested genomic DNA samples used as controls run at different sizes to the long TRF population (ND in Figure 1B). As for the second, different DNA methylation patterns in *tert^+/+^* and mutants could account for the presence of unequal TRF populations due to restriction enzyme sensitivity. To exclude this possibility, we used a restriction enzyme insensitive to DNA methylation, Tru9I, and observed an equivalent TRF pattern (data not shown).

We favour the third hypothesis in which high molecular weight TRFs are derived from cells with longer telomeres present in the same tissue. This is probably the case for testes. Mature sperm from the same individual exhibited only the shorter TRF population, whereas whole testes tissue possesses both. Thus, cells that comprise the testes, other than sperm, must have longer telomeres and are maintained through time by telomerase. In contrast to mature sperm, blood cells isolated from WT individuals exhibited exclusively long telomeres. Consistent with other tissues, *tert^−/−^* zebrafish also have shorter telomeres in blood.

The hypothesis that tissues harbour cells with telomeres of different lengths is corroborated by our telomere PNA-FISH data. *tert^+/+^* tissues, such as the gut, have cells with very different telomere intensities suggestive of shorter and longer telomeres. In contrast, *tert^−/−^* tissues show more homogeneous, low intensity, telomere-FISH. Notably, the majority of high-telomere-intensity cells in *tert^+/+^* are located to the villus proliferative zone. These cells are generally absent in the *tert^−/−^* tissues, suggesting that cells with longer telomeres are strictly telomerase dependent. This suggests that telomerase is activated during embryogenesis and elongates telomeres in stem and early precursor cells. In *tert^−/−^* mutants this cannot happen and, even though cells proliferate initially, their telomeres shorten with each cell division giving rise to populations with shorter telomeres.

Together, our data support the idea that telomerase is limiting for zebrafish telomere maintenance, since we observe long and short telomeres in WT. Accordingly, shorter telomeres in *tert^+/+^* are equivalent in size to the ones present in *tert^−/−^* zebrafish mutants. Shorter telomeres decline rapidly in the first three months of life both in *tert^+/+^* and mutant fish, steadily decreasing for the next three to six months. This period of fast telomere shortening correlates with intense body growth preceding sexual maturity, was also observed in humans [47].

Our work describes a choreography of defects caused by telomere shortening in different tissues over-time. Consistent with the late generation telomerase KO mice, *tert^−/−^* zebrafish die prematurely due to an acute depletion of proliferating cells [30]. The choice between senescence and apoptosis in different cells may dictate the homeostatic threshold of individual tissues. This regulation will determine how the whole organism responds to telomere dysfunction. Proliferative tissues, such as testes, gut and kidney marrow are affected first. Progressive gut degeneration, with severe necrotizing enteritis, is the most likely cause for body wasting and premature death. We observed different apoptotic and senescent responses in different tissues. Immunofluorescence data identified an acute apoptotic response in proliferative tissues, namely the testes and gut and, to a lesser extent, in head kidney. This correlated with increased p53 levels and *puma* expression measured by RT-qPCR, particularly evident in proliferative tissues, such as the gut and testes. This increase in apoptosis is largely cleared at later time points, where DNA damage and senescence became the dominant phenomena in degenerated *tert^−/−^* tissues.

Interfering with *tp53* function in *tert^−/−^tp53^−/−^* double mutants significantly increased proliferation in testes and gut, partially rescuing degenerative phenotypes. This indicates that p53 is a crucial mediator of the *tert^−/−^* degenerative phenotypes in proliferative tissues. Rescue of cell proliferation is not without consequence, since cells continue to accumulate 53BP1 DDR markers and enter apoptosis. However, the apoptotic response in *tert^−/−^tp53^−/−^* zebrafish differs from the *tert/tp53* KO mice [44], [48], since apoptosis increases in comparison to *tert* ^−/−^ single mutants, whereas it decreases in mice. Increase in apoptosis in *tert^−/−^tp53^−/−^* zebrafish is mediated via p53-independent mechanisms, such as those under the control of p63 or p73 [14]. This suggests different mechanistic responses in zebrafish to telomere dysfunction to those in present in the mouse. It also reinforces the idea of separate homeostatic thresholds in different tissues, since p53 removal can only partially rescue testes integrity whereas gut villi length is completely restored in *tert^−/−^tp53^−/−^*.

In conclusion, zebrafish is a suitable model system to understand the effects of telomere shortening during lifespan of an organism. Telomerase is also required for low-proliferative tissue homeostasis. Whether this occurs in an autonomous or non cell-autonomous manner remains to be clarified. Nevertheless, *tert^−/−^* also have shorter telomeres in low-proliferative tissues, suggesting that both scenarios are possible. Reduced or absent telomerase may trigger whole body degeneration by blocking cell proliferation causing an unbalanced tissue homeostasis, and this is largely p53-mediated in high proliferating tissues.

## Materials and Methods

### Ethics statement

All Zebrafish work was conducted according to National Guidelines and approved by the Ethical Committee of the DGV (Portuguese Veterinary Authority) and the European Union Regulatory Agency.

### Zebrafish lines and maintenance

Zebrafish were maintained in accordance with Institutional and National animal care protocols. The telomerase mutant line *tert ^AB/hu3430^* possesses a T→A point-mutation in the *tert* gene. Zebrafish mutant lines were generated by N-Ethyl-N-nitrosourea (ENU) mutagenesis (Utrecht University, Netherlands) [49]. Briefly, adult male zebrafish were randomly mutagenized with ENU and outcrossed against wild-type females. A library of F1 animals was then constructed. Genomic DNA of these F1 animals was isolated and arrayed in PCR plates. The DNA was screened for mutations in target genes by re-sequencing or TILLING. Animals with interesting mutations were recovered from the library (either re-identified from a pool of living F1 fish or recovered by *in vitro* fertilization with frozen sperm) and outcrossed against wild-type fish.

*tert ^AB/hu3430^* line is available at the ZFIN repository (ZFIN ID: ZDB-GENO-100412-50) from the Zebrafish International Resource Center (ZIRC) and was generously provided to us by Dr. L. Bally-Cuif at the Zebrafish Neurogenetics Department, German Research Center for Environmental Health. The *tert^AB/hu3430^* used in this paper was subsequently outcrossed 5 times with WT AB for clearing of potential background mutations derived from the random ENU mutagenesis from which this line was originated. The *tert^hu3430/hu3430^* homozygous mutant is referred in the paper as *tert^−/−^* and was obtained by incrossing our *tert^AB/hu3430^* strain. Genotyping was performed by PCR of the *tert* gene (Table S1) followed by sequencing.

Overall characterization of *tert^−/−^* zebrafish was performed in F1 and F2 animals produced by *tert^+/−^* incross. All 1, 3 and 6 months analysis refers to F1 animals and 12 months analysis refers to F2. The premature death phenotype depicted in Figure 2C refers to F1 animals only

### Telomerase activity assay (TRAP)

Telomerase activity was measured using the TRAPEZE Telomerase Detection Kit (S7700, Millipore, MA, USA) as described by manufacturers. Briefly, fish were sacrificed in 200 mg/L of MS-222 (Sigma, MO, USA) and a small portion of skin and fin were extracted from at least three different individuals of the different genotypes. Protein extracts were prepared by mashing tissue sections on ice with a micro-pestle in a 1.5 ml eppendorf tube, in 100 µl of CHAPS buffer with proteinase inhibitors cocktail (Sigma, MO, USA) and RNase inhibitor (200 U/ml, Invitrogen, UK). Cell extracts were incubated on ice for 30 minutes and centrifuged at 12,000×g for 20 minutes at 4°C. The supernatant was quantified using Bradford protein quantification reagent (Pierce, IL, USA) and the TRAP assay was performed using 2 µg of protein/sample. The positive control was provided by the TRAP kit and used as described (S7700, Millipore, MA, USA).

### Telomere restriction fragment (TRF) analysis by Southern blot

TRF analysis was performed as previously described [50]. Briefly, genomic DNA extraction from freshly isolated tissue was performed using extraction buffer (50 mM Tris-HCl pH 8; 50 mM EDTA; 10 mM NaCl; 1% SDS) supplemented with 1 mg/ml Proteinase K (Sigma, MO, USA) and RNase A (1∶100 dilution, Sigma, MO, USA) prior to use. Samples were incubated at 50°C for 18 h in a thermomixer and genomic DNA was extracted by equilibrated phenol-chloroform (Sigma, MO, USA) and chloroform-isoamyl alcohol extraction (Sigma, MO, USA). Blood genomic DNA was extracted with TNES buffer (10 mM Tris pH 7.4; 100 mM NaCl; 10 mM EDTA; 0.5% SDS), supplemented with RNase A (1∶100 dilution, Sigma, MO, USA) prior to use. Samples were incubated 10 minutes at RT and extracted as for other tissues. Genomic DNA was quantified and normalized so the same amount of DNA was digested with RSAI and HINFI enzymes (NEB, MA, USA) as described previously [50] for 12 h at 37°C. BAL31 (NEB, MA, USA) digestion was performed at 30°C for different time points. Samples were ran on a 20 cm 0.6% agarose gel, in 0.5% TBE buffer, at 4°C for 17 h at 110 constant voltage. Southern blotting was performed as previously described [51].

### Histological preparation and phenotypic analysis

Fish were sacrificed as described above, fixed overnight in 4% paraformaldehyde and decalcified in 0.5 M EDTA for 24–48 h at 4°c. Whole fish were then paraffin-embedded and 5 micrometer sections were stained with Hematoxylin-Eosin for histopathological analysis. Embryos were fixed overnight in 4% PFA at 4°C as above and then placed in 100% methanol at 4°C before processing. After several washes in PBS (phosphate buffer saline) the embryos were cryoprotected on a sucrose 15%/PBS solution and then embedded in 7,5% pork skin gelatine (Sigma)/15% sucrose/PBS for one hour at 37°C. The 1 cm^2^ blocks were frozen in isopenthane/liquid nitrogen and stored at −80°C until sectioning. The embedded samples were cut in 12 um sections with a cryostat (Leica CM 3050S). Hematoxilin-Eosin staining was performed in serial cuts.

### Immunofluorescence (IF) and confocal analysis

Whole fish slides were sub-boiled for 10 minutes at 800 W in a microwave in citrate buffer (10 mM Sodium Citrate, pH 6) for antigen retrieval. Slides were washed 3 times in dH20 for 5 minutes each, followed by TBST (Tween 0.1%) for 5 minutes. After washes, slides were blocked for 1 hour at RT in 0.25% BSA in PBST (Triton 0.3%). The following primary antibodies were used: rabbit monoclonal antibodies against Proliferation Cell Nuclear Antigen (PCNA, Santa Cruz, CA, USA, 1∶50 dilution), 53BP1 (Life-span Biosciences, WA, USA, 1∶100) and anti-PLZF (Life-span Biosciences, WA, USA, 1∶100). Incubation with primary antibodies was performed overnight in the dark, at 4°C, followed by 5 minutes, 1 hour and two 10 minutes PBS washes. Secondary antibody Alexa Fluor 568 goat anti-rabbit (Invitrogen, UK, 1∶500 dilution) was then applied overnight at 4°C, followed by final washes in PBS, as described for the primary antibody washes. Apoptosis was detected using the *In Situ* Cell Death Detection Kit (Roche, SW) according to manufacturer's instructions. Briefly, after de-parafinization, slides were incubated with 40 µg/ml Proteinase K in 10 mM Tris-HCl pH 7.4, 30 minutes at 37°C. Slides were washed in 2×5 minutes in PBS and then incubated with TUNEL labelling mix (protocol indicated by the supplier). Washes were performed as previously described. Slides were incubated with TO-PRO3 (Molecular Probes, Invitrogen, UK, 1∶5000 dilution) and DAPI (Sigma, MO, USA, 1∶2000 dilution) nuclear staining for 30 minutes at room temperature in the dark, followed by two 5 minutes' washes with PBS. Coverslips were then mounted with DAKO Fluorescence Mounting Medium (Sigma, MO, USA). Confocal images were acquired on Leica TCS SP5 II (Leica Microsystems, GER) equipped with Leica Las AF Lite software and with appropriate configurations for multiple colour acquisition. For quantitative and comparative imaging, equivalent image acquisition parameters were used.

### Telomere PNA-FISH

Immuno-FISH was performed as in [39]. Briefly, zebrafish paraffin sections, processed as for IF were hydrated by incubation in 100% Histoclear, 100, 95 and 70% methanol for 5 min and in distilled water for 5 min. Whole fish slides were sub-boiled for 10 minutes at 800 W in a microwave in citrate buffer (10 mM Sodium Citrate, pH 6) for antigen retrieval. After cooling the slides were washed with distilled water for 5 min (2×). Slides were washed three times in PBS and dehydrated with 70, 90 and 100% ethanol for 3 min each. Sections were denatured for 5 min at 80°C in hybridization buffer (70% formamide (Sigma), 25 mM MgCl_2_, 1 M Tris pH 7.2, 5% blocking reagent (Roche)) containing 2.5 µg/ml Cy-3-labelled telomere specific (CCCTAA) peptide nuclei acid probe (Panagene), followed by hybridization for 2 h at room temperature in the dark. The slides were washed twice with 70% formamide in 2×SSC for 15 minutes, followed by 10 minutes wash with 2×SSC and PBS. Sections were incubated with DAPI (SIGMA), mounted and imaged. Z stacking was performed (a minimum of 40 optical slices with ×100 objective) followed by Image-J deconvolution.

### Senescence-associated β-galactosidase assay

β-galactosidase assay was performed as previously described [25]. Briefly, sacrificed zebrafish adults were fixed as before and then washed 3 times for 1 h in PBS-pH 7.4 and for a further 1 h in PBS-pH 6.0 at 4°C. β-galactosidase staining was performed for 24 h at 37°C in 5 mM potassium ferrocyanide, 5 mM potassium ferricyanide, 2 mM MgCl_2_ and 1 mg/ml X-gal, in PBS adjusted to pH 6.0. After staining, fish were washed 3× for 5 minutes in PBS pH 7 and processed for de-calcification and paraffin embedding as before. Sections were stained with hematoxilin for nuclear detection and images were acquired in a bright field microscope (Leica DMLB2, GER).

### Real-time quantitative PCR

Age- and sex-matched fish were sacrificed in 200 mg/L of MS-222 (Sigma, MO, USA) and portions of each tissue (gonads, gut, liver, head kidney and muscle) were retrieved and immediately snap-frozen in liquid nitrogen. RNA extraction was performed in TRIzol (Invitrogen, UK) by mashing each individual tissue with a pestle in a 1.5 ml eppendorff tube. After incubation at RT for 10 minutes in TRIzol, chlorophorm extractions were performed. Quality of RNA samples was assessed through BioAnalyzer (Agilent 2100, CA, USA). Transcription into cDNA was performed using random primers (20 µg) (Promega C1181, WI, USA). Quantitative PCR (qPCR) was performed using PerfeCTa SYBR Green FastMix, ROX (Quanta, MD, USA) and an ABI 7900HT Sequence Detection System (Applied Biosystems, CA, USA). qPCRs were carried out in triplicate for each cDNA sample. Relative mRNA expression was normalized to *beta-actin* and *rpl13* α (data not shown) mRNA expression using the ΔCT method (derived from the *Livak & Schmittgen* method, 2001). Primer sequences are listed in Table S1.

### Immunoblot analysis

Each tissue was dissected and homogenized in HEPES buffer (HEPES 10 mM, KCl 300 mM, MgCl2 3 mM, CaCl2 100 mM, Triton X-100 0.45%, Tween-20 0.05%, pH 7.6) including complete protease inhibitor cocktail (Roche Diagnostics). Cell extracts were incubated on ice and centrifuged at 13,000 rpm for 10 minutes at 4°C. The supernatant was collected and quantified using Bradford protein quantification reagent (Pierce). Loading mix was added to protein extracts, heated at 95°C for 5 minutes and loaded onto a 12.5% SDS-PAGE gel (80 µg of protein/sample).

After electroblotting the gel onto a PVDF membrane, incubation was performed overnight at 4°C with anti-Tp53 (AnaSpec, 55342) specific for zebrafish, in TBS-T with 5% milk powder (using a 1∶300 dilution). Chemiluminescence detection was performed with an ECL KIT (Amersham).

Western blots were performed on WT and tert−/− 10 month-old tissue samples (N = 3–4).

### Doxorubicin assay

To induce a DNA-damage p53-dependent response, adult fish were anesthetized in MS-222 (Sigma) and doxorubicin (Sigma) was injected intraperitoneally. A dosage of 15 mg/kg body weight was used. Fish were allowed to recover for 24 h, after which they were sacrificed in 200 mg/L of MS-222 (Sigma) and the organs collected for analysis of p53 protein levels by western blot.

### Statistical analysis

#### Immunofluorescence

Image edition was performed in Adobe Photoshop CS5.1. All statistical analysis was performed in *GraphPad Prism5*, using Mann Whitney's unpaired t-test when only two points were compared. More than one point comparison over-time was performed by two-way ANOVA test with Bonferroni post-correction. A critical value for significance of p<0.05 was used throughout the study.

#### Real-time quantitative PCR

Statistical analysis was performed in *GraphPad Prism5*, two-way ANOVA with Bonferroni post-correction. A critical value for significance of p<0.05 was used throughout the study.

## Supporting Information

Figure S1 *tert^−/−^* zebrafish have shorter telomeres than *tert^+/+^* in all tissues tested. A) Representative southern blots and TRF analysis of different tissues at the age of 3 months, show decreased telomere sizes in *tert^−/−^* as compared to *tert^+/+^* siblings. Note that all tissues have both long and short TRF populations in the *tert^+/+^*, except the blood, where only a long TRF of approximately 15 Kb is detected. *tert^−/−^* show a severe decrease of these long telomeres, and mainly show the short TRF smear of approximately 6 Kb. B) Mean TRF peak quantifications of the southern blot shown in A). C) Representative southern blot and TRF analysis of Bal31 (a 5′ and 3′ terminal exonuclease) restriction of fin genomic DNA shows that all telomeric signals correspond to terminal sequences. N≥3. Data are represented as mean +/− SEM.
(TIF)

Figure S2 First generation *tert^−/−^* show premature male infertility. A) *tert^−/−^* mutant males are infertile by 6 months of age, represented here as percentage of non-fertilized eggs per cross (Mean nr. of non-fertilized eggs/total number of eggs produced by the female). Number of crosses = 3. B). The few F1 maternal zygotic progeny are not viable due to gross abnormalities during embryonic development. N≥3. Data are represented as mean +/− SEM.
(TIF)

Figure S3 *tert^−/−^* proliferative tissues accumulate strong 53BP1 foci. Panel showing representative images of cells in testis and gut of wild-type and *tert^−/−^* fish at c.12 months of age. *tert* ^−/−^ tissues accumulate cells presenting strong 53BP1 foci, as highlighted by the yellow arrows, compared to a more diffuse 53BP1 staining in most wild-type cells.
(TIF)

Table S1 List of primers used in RT-qPCR expression analysis and *tert* genotyping.
(DOC)

Table S2 Time-dependent histopathological changes in *tert^−/−^* zebrafish. Semi-quantitative histopathological analysis was performed using a score ranging from (−) to (+++), depending on the severity and extent of the lesions: (−) none, (+) minimal to mild, (++) moderate, (+++) severe.
(DOC)
