# Correction: Telomerase Is Required for Zebrafish Lifespan

**DOI:** 10.1371/journal.pgen.1006652

**Published:** 2017-03-13

**Authors:** Catarina M. Henriques, Madalena C. Carneiro, Inês M. Tenente, António Jacinto, Miguel Godinho Ferreira

In panel A of Fig 1, a duplication of the *tert*^*−/−*^ Skin lane appears where the *tert*^*−/−*^ Fin lane should be. Please view the correct Fig 1 here with the correct *tert*^*−/−*^ Fin lane shown. Further clarification and copies of the original gels can be found in S1 File.

**Fig 1:**
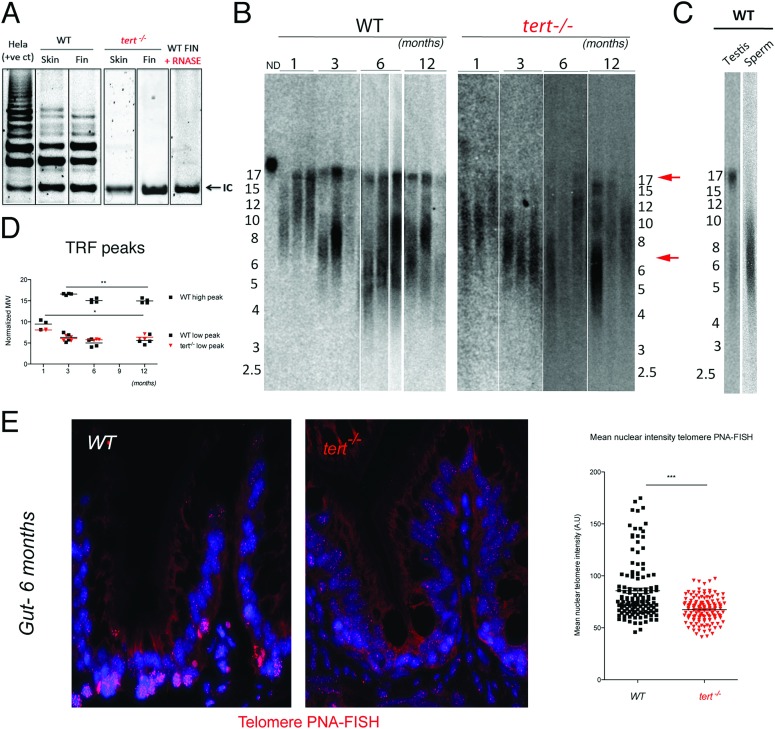
Telomerase mutant zebrafish have shorter telomeres than WT siblings. A) Representative image of TRAP assay showing that telomerase is not active in the *tert*^*−/−*^ zebrafish, as compared to *tert*^*+/+*^ siblings. Here shown are caudal fin and skin protein extracts. Hela cell extract is shown as positive control. N = 4. B) Representative image of restriction fragment analysis of caudal fin genomic DNA of 3 different individuals at different ages, by southern blot (random primer-labelled telomeric probe (CCCTAA)_12_^32^P-dCTP). *tert*^*+/+*^ Zebrafish have heterogeneous telomeres, with two distinct peaks of different lengths. In *tert*^*+/+*^ the highest peak (∼16 Kb, top red arrow) becomes more distinct after 1 months of age and decreases in length over-time (B and D). The lowest peak of telomere intensity also decreases in length (bottom red arrow, B and D). *tert*^*−/−*^ zebrafish have shorter telomeres than *tert*^*+/+*^ siblings in different tissues (see also Figure S1A and S1B), observed by the decrease in length of the higher TRF peak. The shortest TRF peaks accompany those of *tert*^*+/+*^ siblings, and decrease over-time at similar rates. C) Testes fractionation in *tert*^*+/+*^ reveals the two-telomere length populations in whole testes, whereas mature sperm only shows the shorter TRF smear of about 6 Kb, suggesting different telomere lengths in different cells within a tissue. D) TRF mean sizes were calculated as described in [50]. E) Telomere PNA-FISH in 6-month-old gut tissue shows cells with different telomere intensities in the wild type, mainly localizing to the proliferative niche. In contrast *tert*^*−/−*^ mutants display cells with less bright and more homogeneous telomere intensity.

## Supporting information

S1 File Correction and copies of the original gel scans from where lanes were selected. (DOCX)
