# Supplementary figures and images for: Telomerase Is Required for Zebrafish Lifespan
Source: PLoS Genet. 2013 Jan 17;9(1):e1003214. doi: 10.1371/journal.pgen.1003214 (PMC3547866; doi:10.1371/journal.pgen.1003214)

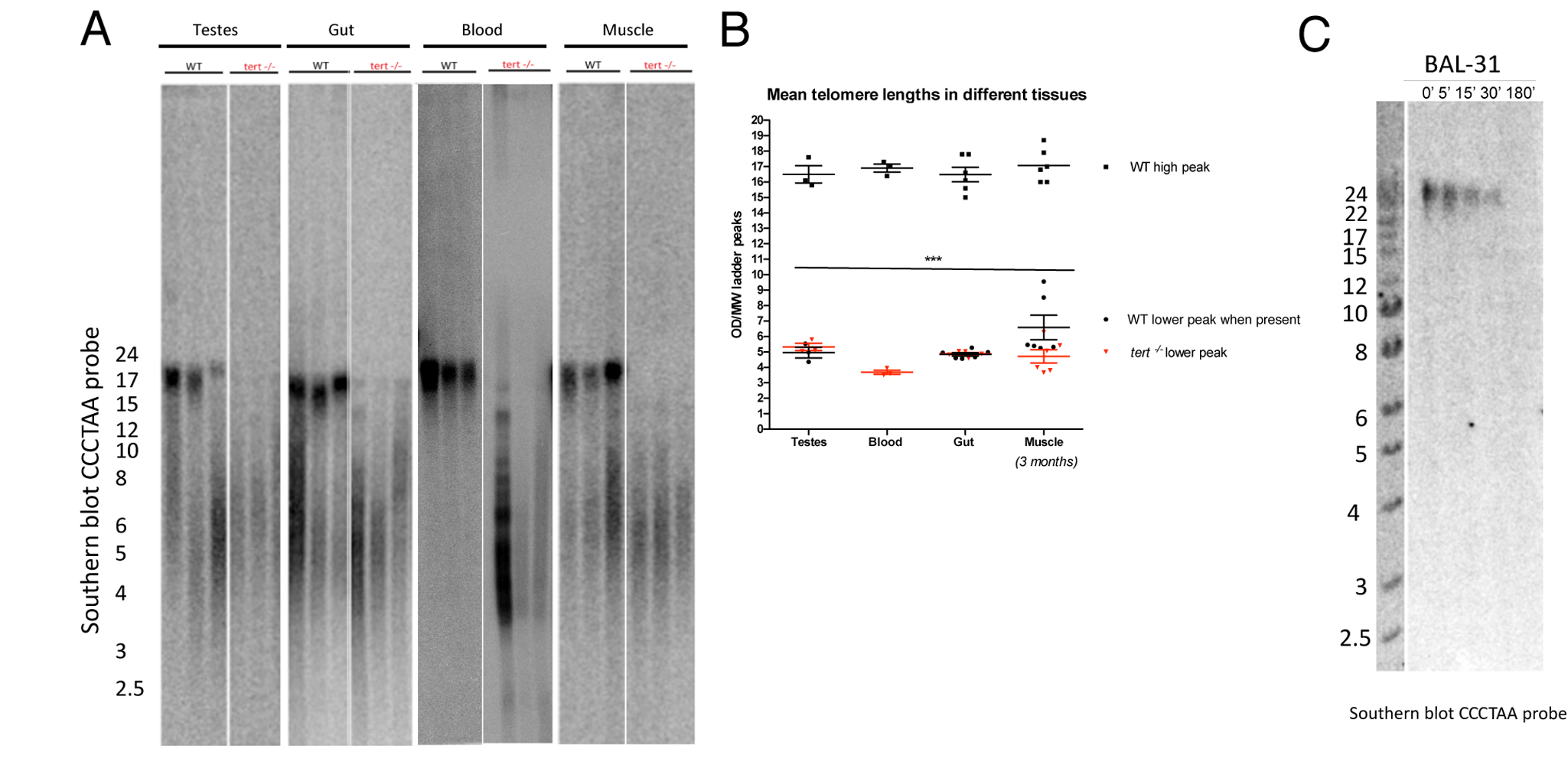

Supplement: Figure S1 — tert−/− zebrafish have shorter telomeres than tert+/+ in all tissues tested. A) Representative southern blots and TRF analysis of different tissues at the age of 3 months, show decreased telomere sizes in tert−/− as compared to tert+/+ siblings. Note that all tissues have both long and short TRF populations in the tert+/+, except the blood, where only a long TRF of approximately 15 Kb is detected. tert−/− show a severe decrease of these long telomeres, and mainly show the short TRF smear of approximately 6 Kb. B) Mean TRF peak quantifications of the southern blot shown in A). C) Representative southern blot and TRF analysis of Bal31 (a 5′ and 3′ terminal exonuclease) restriction of fin genomic DNA shows that all telomeric signals correspond to terminal sequences. N≥3. Data are represented as mean +/− SEM. (TIF) [file pgen.1003214.s001.tif]

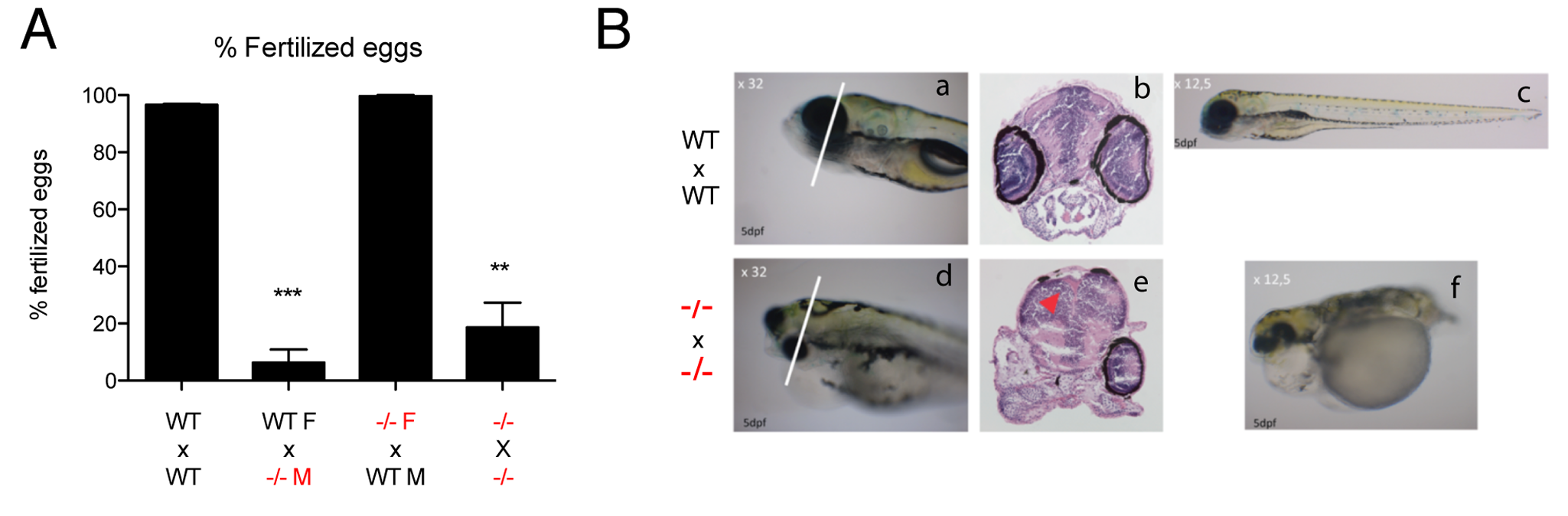

Supplement: Figure S2 — First generation tert−/− show premature male infertility. A) tert−/− mutant males are infertile by 6 months of age, represented here as percentage of non-fertilized eggs per cross (Mean nr. of non-fertilized eggs/total number of eggs produced by the female). Number of crosses = 3. B). The few F1 maternal zygotic progeny are not viable due to gross abnormalities during embryonic development. N≥3. Data are represented as mean +/− SEM. (TIF) [file pgen.1003214.s002.tif]

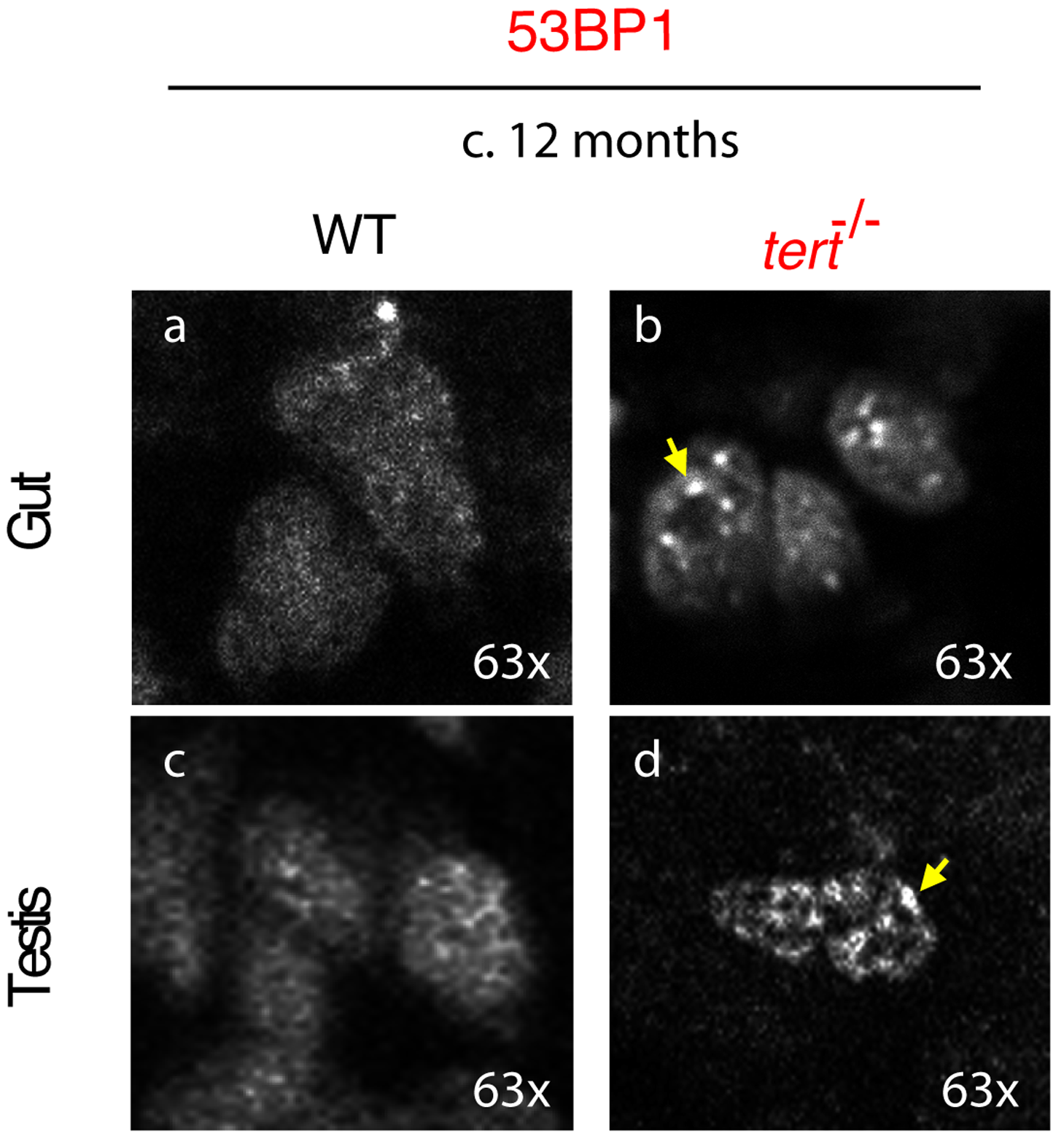

Supplement: Figure S3 — tert−/− proliferative tissues accumulate strong 53BP1 foci. Panel showing representative images of cells in testis and gut of wild-type and tert−/− fish at c.12 months of age. tert −/− tissues accumulate cells presenting strong 53BP1 foci, as highlighted by the yellow arrows, compared to a more diffuse 53BP1 staining in most wild-type cells. (TIF) [file pgen.1003214.s003.tif]
