# Supplementary material for: Telomerase Is Required for Zebrafish Lifespan
Source: PLoS Genet. 2013 Jan 17;9(1):e1003214. doi: 10.1371/journal.pgen.1003214 (PMC3547866; doi:10.1371/journal.pgen.1003214)
Supplement: Table S1 — List of primers used in RT-qPCR expression analysis and tert genotyping. (DOC) [file pgen.1003214.s004.doc]

| **Gene name** | **Primer sequences** | **Reference** |
| --- | --- | --- |
| *beta-actin* | forward – 5’ TTCACCACCACAGCCGAAAGA 3’  reverse – 5’ TACCGCAAGATTCCATACCCA 3’ |  |
| *cdkn1a* | forward – 5’ ATGCAGCTCCAGACAGATGA 3’  reverse – 5’ CGCAAACAGACCAACATCAC 3’ | Duan et al., 2011 |
| *cyclin G1* | forward – 5’ GTGATGAAGATTCAGCCCAAGC 3’  reverse – 5’ CACTGGCCAGAGGGACATTTTTCT 3’ | Our study |
| *puma* | forward – 5’ CCTCACATGATGCCTTCAGC 3’  reverse – 5’ CATTGATGGTGTCCGAGACC 3’ |  |
| *tp53* | forward – 5’ GGTGCTGAATGGACAACTGTGCT 3’  reverse – 5’ GCAACTGACCTTCCTGAGTCTCC 3’ | Our study |
| *tert* | forward – 5’ GACGACCAGTTCGGATCCCTTC 3’  reverse – 5’ CTTTACCCTCCGCCGCTTTACC 3’ | Our study |

C.Henriques et al._ Sup_Tabl1
