# Supplementary material for: Telomerase Is Required for Zebrafish Lifespan
Source: PLoS Genet. 2013 Jan 17;9(1):e1003214. doi: 10.1371/journal.pgen.1003214 (PMC3547866; doi:10.1371/journal.pgen.1003214)
Supplement: Table S2 — Time-dependent histopathological changes in tert−/− zebrafish. Semi-quantitative histopathological analysis was performed using a score ranging from (−) to (+++), depending on the severity and extent of the lesions: (−) none, (+) minimal to mild, (++) moderate, (+++) severe. (DOC) [file pgen.1003214.s005.doc]

|  | **3 month-old** | **6 month-old** | **c. 12 month-old** |
| --- | --- | --- | --- |
| **Testes** | +++ | n.a. | +++ |
| **Liver** | ++ | ++ | ++ |
| **Gut** | - | + | +++ |
| **Kidney** | - | ++ | +++ |
| **Gills** | - | - | + |
| **Bone marrow** | - | - | ++ |
| **Pancreas** | - | n.a. | ++ |
