# Supplementary material for: Correction: Telomerase Is Required for Zebrafish Lifespan
Source: PLoS Genet. 2017 Mar 13;13(3):e1006652. doi: 10.1371/journal.pgen.1006652 (PMC5347992; doi:10.1371/journal.pgen.1006652)
Supplement: S1 File — (DOCX) [file pgen.1006652.s001.docx]

For clarification, please find below the correction and copies of the original gel scans from where lanes were selected.


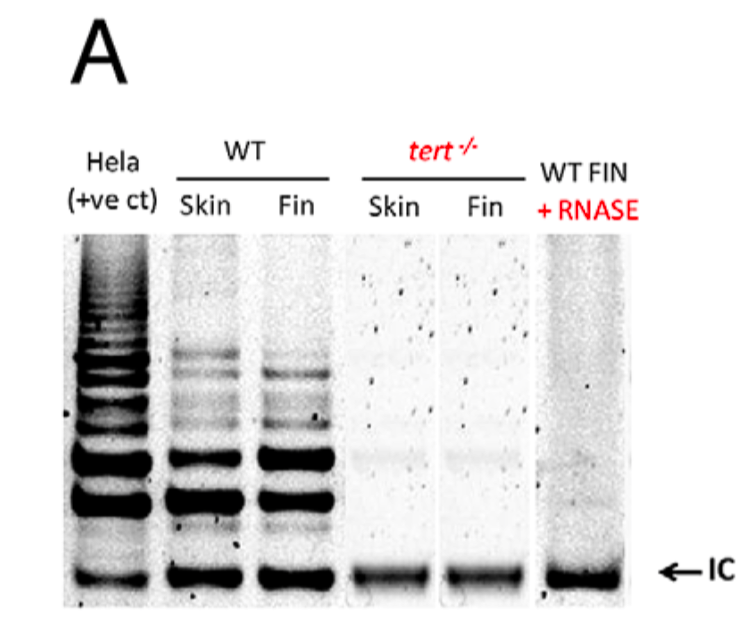


Published Figure 1A with the accidentally duplicated *tert^-/-^* Skin lane instead of what should have been the fin lane. See below for correction:

CORRECTED Figure 1A with the correct *tert*^-/-^ Fin lane


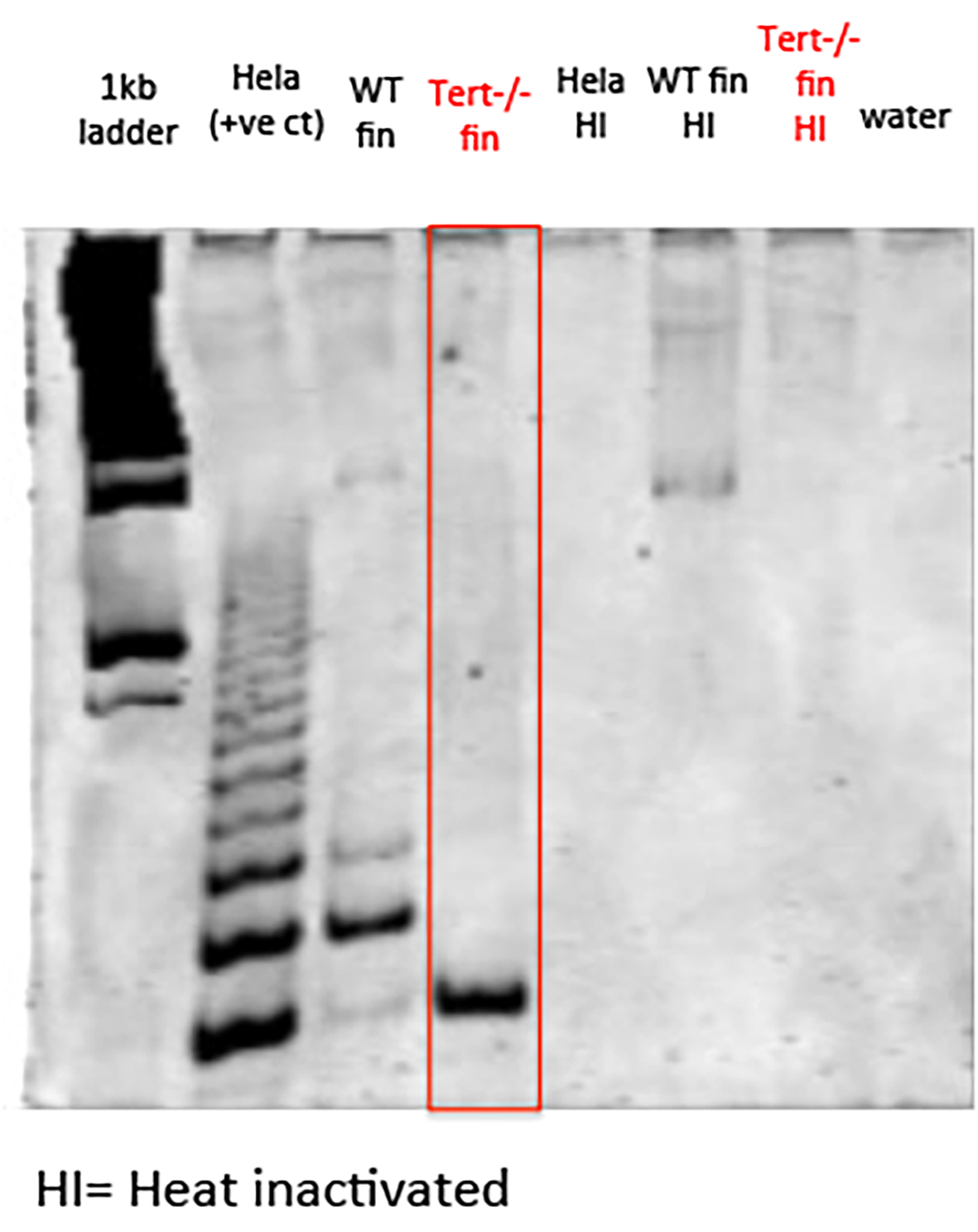

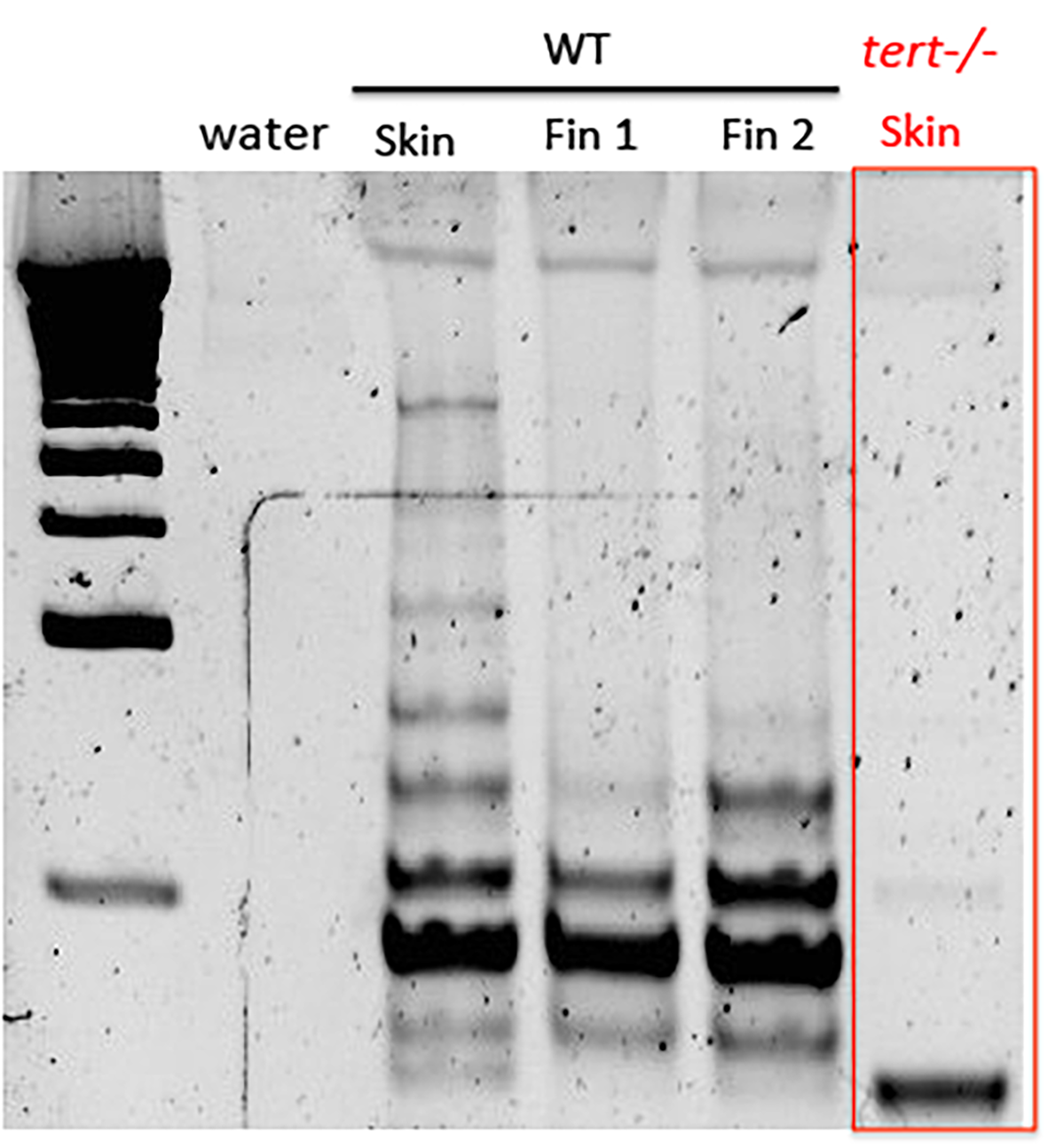

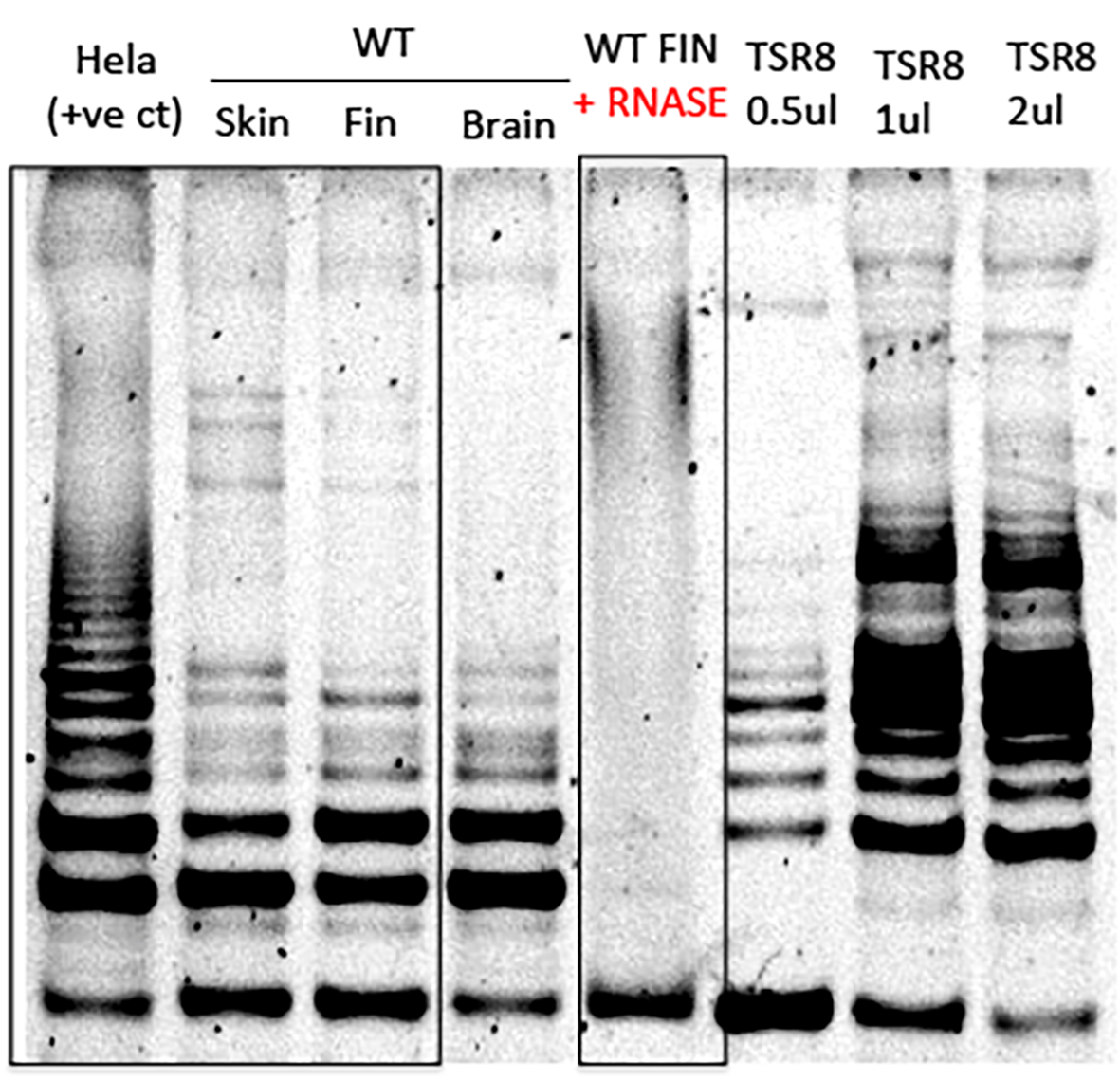


50bp

ladder

Original TRAP gel scans from which the representative lanes of multiple repeats in the lab were selected:


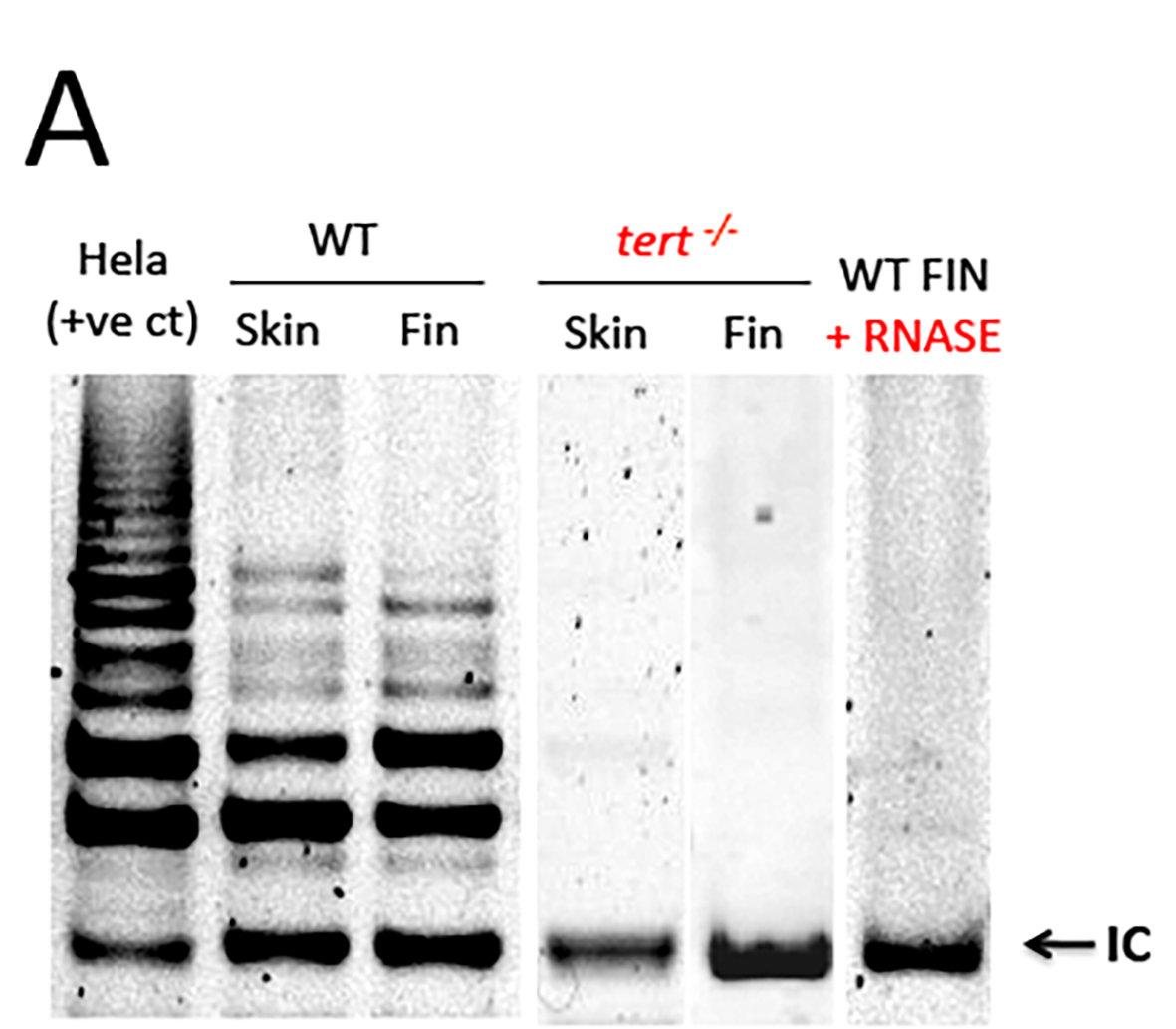


Final Corrected TRAP

Figure 1A) **correction in Bold.** Telomerase mutant zebrafish have shorter telomeres than WT siblings. A) Representative image of TRAP assay showing that telomerase is not active in the *tert^-/-^* zebrafish, as compared to *tert^+/+^* siblings. Here shown are caudal fin and skin protein extracts. Hela cell extract is shown as positive control. N=4. **Lanes run on different gels are indicated by dividing black boxes.**
